# Synthesis of long and functionally active RNAs facilitated by acetal levulinic ester chemistry

**DOI:** 10.1093/nar/gkaf1525

**Published:** 2026-01-19

**Authors:** Zidi Lyu, Adam Katolik, Iqra Yaseen, Adrian A Pater, Francis Robert, Sidong Huang, Keith T Gagnon, Peter J Unrau, Masad J Damha

**Affiliations:** Department of Chemistry, McGill University, Montreal, Quebec H3A 0B8, Canada; Department of Chemistry, McGill University, Montreal, Quebec H3A 0B8, Canada; Department of Molecular Biology and Biochemistry, Simon Fraser University, Burnaby, British Columbia V5A 1S6, Canada; Department of Biochemistry, Wake Forest University School of Medicine, Winston-Salem, NC 27157, United States; Department of Biochemistry, Rosaline & Morris Goodman Cancer Institute, McGill University, Montreal, Quebec H3G 1Y6, Canada; Department of Biochemistry, Rosaline & Morris Goodman Cancer Institute, McGill University, Montreal, Quebec H3G 1Y6, Canada; Department of Biochemistry, Wake Forest University School of Medicine, Winston-Salem, NC 27157, United States; Department of Molecular Biology and Biochemistry, Simon Fraser University, Burnaby, British Columbia V5A 1S6, Canada; Department of Chemistry, McGill University, Montreal, Quebec H3A 0B8, Canada

## Abstract

Recent advances in RNA-based therapeutics have created a demand for synthetic RNAs that are 100 nucleotides (nts) or longer. In this study, we present the use of 2′-acetal levulinic ester (2′-ALE) phosphoramidites for the synthesis of long RNAs that are at least 215 nts in length. We have developed protocols for rapid (2–4 min) and efficient coupling (>99%) of 2′-ALE monomers and established a rapid, on-column deprotection of RNA strands requiring short alkylamine treatments at room temperature. The results of these studies enabled the successful syntheses of sgRNAs (99 nt), sgRNA tagged with fluorogenic Mango II and Broccoli aptamers (130–170 nt), and 5′-capped minimal mRNAs (200–215 nt), each exhibiting robust functional activity in both cell-free and cellular systems. We also found that the incorporation of 2′-*O*-methyl-adenosine in the poly(A) tail of synthetic mRNAs markedly enhanced protein expression, highlighting the ALE platform’s compatibility for systematic exploration of RNA chemical diversity. Collectively, these results establish 2′-ALE chemistry as a promising platform for the synthesis of long and functionally active RNAs.

## Graphical Abstract

**Graphical Abstract:**
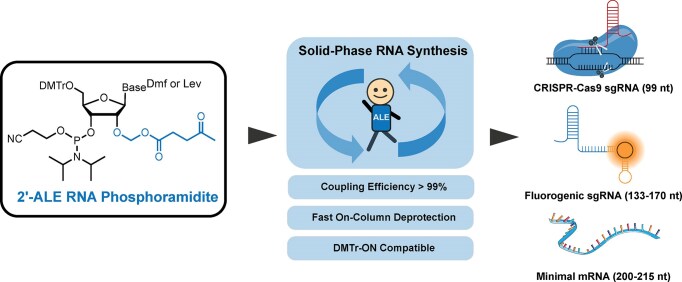

## Introduction

Solid-phase oligonucleotide synthesis (SPOS) enables high-fidelity sequence control and precise, site-specific incorporation of both natural and artificial modifications [1]. However, yields produced by SPOS decrease significantly as the length of the oligonucleotide increases due to the difficulty of ensuring complete reactions over many synthesis cycles. Recent advances in nucleic acid-based therapeutics have created a demand for long chemically synthesized RNAs, exceeding the length of those currently approved. For example, single-guide RNAs (sgRNAs) used in standard CRISPR-Cas9 systems are typically around 100 nt long [2, 3], while prime editing guide RNAs (pegRNAs) are generally longer, often falling between 120 and 145 nts, but may extend to 170–190 nts [4, 5]. Length limitations and the ability to introduce chemical modifications are two primary reasons driving the use of chemical synthesis for shorter sgRNAs, while longer RNAs such as pegRNA and mRNA are typically produced by *in vitro* transcription (IVT) and/or ligation approaches [6, 7].

Relative to DNA synthesis, the chemical synthesis of RNA is complicated by an additional protecting group for the reactive 2′-hydroxyl on the ribose sugar that must be stable throughout chain assembly and readily removed under conditions that will not lead to degradation of the RNA. Thus, efficacy of RNA synthesis hinges profoundly on the selection of protecting groups onto nucleoside 3′-*O*-phosphoramidites at the 2′-hydroxyl group and nucleobase positions. The 2′-*O*-*tert*-butyldimethylsilyl (TBDMS) protecting group, as adapted by Ogilvie and coworkers for RNA synthesis, is the gold standard in the field because it is stable under various conditions and can be removed safely and efficiently using fluoride [*e.g*. tetra-*n*-butylammonium fluoride (TBAF) or triethylamine trihydrofluoride (TREAT-HF)] at the end of the synthesis [8–13]. TBDMS chemistry was crucial in the synthesis of Patisiran, the first siRNA (small interfering RNA) approved for clinical use, and continues to be the chemistry of choice for RNA synthesis [14]. From our experience, solid-phase synthesis of RNA using TBDMS chemistry is quite appropriate for RNA up to 120 nts in length. Going beyond this length is difficult due to 2′-steric crowding and the maximum coupling yields that can be attained with the TBDMS group (ca. 97%–98%). Furthermore, removal of fluoride at the end of synthesis is an extra step that becomes particularly challenging when performing high-throughput RNA synthesis or large-scale production.

**Figure 1.**
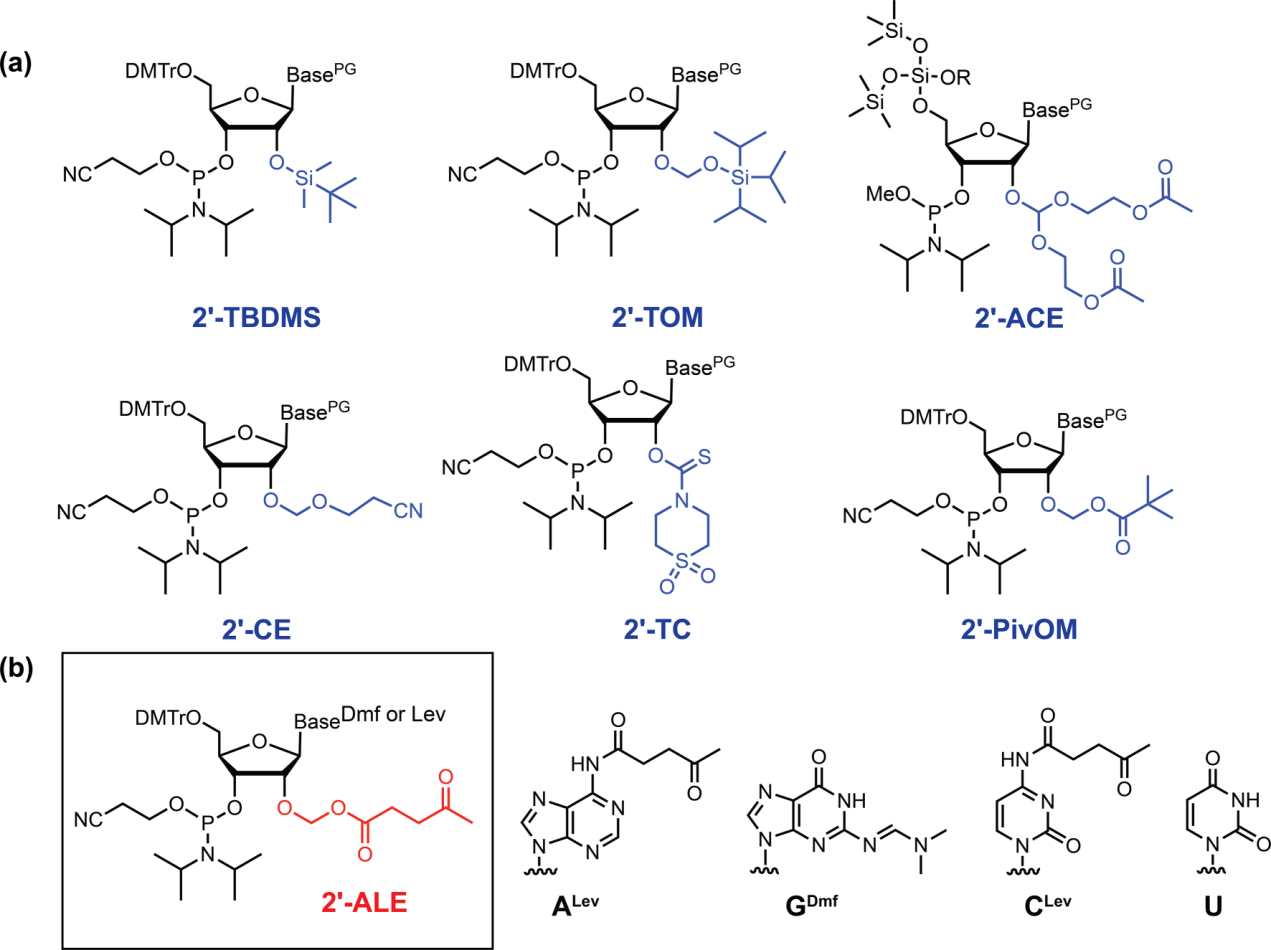
**(a**) Common 2′ protection strategies for RNA solid-phase synthesis. 2′-TBDMS: 2′-*O*-tert-butyldimethylsilyl; 2′-TOM: 2′-*O*-triisopropylsilyloxymethyl; 2′-ACE: 2′-*O*-bis(2- acetoxyethoxy)methyl; 2′-CE: 2′-*O*-cyanoethyl; 2′-TC: 2′-*O*-1,1-dioxo-1λ(6)-thiomorpholine-4-carbothioate; 2′-PivOM: 2′-*O*-pivaloyloxymethyl. (**b**) Structure of 2′-*O*-acetal levulinic ester (2′-ALE) RNA phosphoramidite building block with the base labile protecting group structures. Base^PG^: nucleobases with protecting group; Dmf: dimethylformamidine group; DMTr: 4,4′-dimethoxytrityl; Lev: levulinyl group.

Notwithstanding the widespread use and acceptance of the TBDMS approach, ample other chemistries have been developed and reviewed (Fig. 1a) [15, 16]. The 2′-tris(isopropylsilyl)oxy)methyl (2′-TOM) group is often recommended for longer RNA synthesis because the oxymethyl spacer extends the bulky silyl group further away from the active phosphoramidite center. Coupling yields above 98.5% are consistently achieved with TOM monomers [17]. However, removal of the TOM group still relies on fluoride-mediated cleavage at elevated temperature. The 2′-bis(acetoxyethoxy)methyl (2′-ACE) group offers even higher stepwise yields (>99%), but the synthesis cycle requires HF treatments for 5′-*O* deprotection and necessitates more specialized instrument setup [18]. The 2′-cyanoethoxymethyl (2′-CEM) group enabled the synthesis of 110–170 mer RNAs with excellent overall yield, but the removal of the CEM group does not avoid the additional step of fluoride treatment [19, 20]. To the best of our knowledge, a 170-mer is the longest chemically synthesized RNA using the CEM protection group without enzymatic ligation. Dellinger and coworkers reported the synthesis of a ribozyme (54-mer) [21] and full-length sgRNAs (100-mer) [22] using 2′-*O*-thionocarbamate (2′-TC) protected nucleoside phosphoramidites. The TC group has the advantage that it can be removed by ethylenediamine treatment under the conditions that simultaneously deprotect the nucleobase protecting groups and release the RNA from the solid support by an aqueous wash [21]. Similarly, Debart and coworkers introduced 2′-*O*-pivaloyloxymethyl (2′-PivOM) monomers reporting coupling yields more than 99% [23–25], and deprotection of the RNA requiring concentrated aqueous ammonia (3 h) preceded by a treatment with DBU (1 M in CH_3_CN) to remove the cyanoethyl phosphate protecting groups. Independently, we introduced 2′-*O*-acetal levulinic ester (2′-ALE) monomers (Fig. 1b) for the synthesis of RNA sequences on both microarrays and controlled pore glass (CPG) [26, 27]. In the presence of 4,5-dicyanoimidazole (DCI) as the activator, ALE monomers coupled on microarrays with 99 % (rG, rC) to 99.9 % (rU, rA) efficiencies, like the best results typically obtained in DNA synthesis. After synthesis, the RNA strands were deprotected in a stepwise manner, first with Et_2_NH/ACN (1:9, v/v) for 90 min to remove cyanoethyl groups on phosphates, and then with a buffered solution of 0.5 M hydrazine hydrate in pyridine/AcOH 3:2 (2 h, room temperature (r.t.)) to simultaneously deblock the nucleobases and 2′-OH groups.

Here, we report on the utility of ALE chemistry for generating RNAs from 26 nts up to 215 nts in length with sequences provided in Supplementary Table S1. Deprotection of RNA strands required mild and rapid alkylamine treatments at room temperature (r.t.), suggesting a potentially efficient method for high-throughput RNA synthesis. The results of these studies were applied to the successful syntheses of sgRNAs (99 nt), sgRNA fused with fluorogenic Mango II and Broccoli aptamers (130–170 nt), as well as a series of 5′-capped mRNAs (200–215 nt) that exhibited robust translational activity in both cell-free and cellular systems. This establishes ALE chemistry as a viable platform for routinely synthesizing longer RNA strands.

## Materials and methods

### Oligonucleotide solid-phase synthesis

The HPLC-grade acetonitrile (ACN) was obtained from Thermo Fisher Scientific. A drying trap-XL (ChemGenes DMT-1972) was added to the ACN at least 1 day before synthesis. The Chemical Phosphorylation Reagent II (CPR II) was obtained from Glen Research (Glen Research 10-1901). All remaining reagents were obtained from ChemGenes Corporation, including: (1) DMTr Removal Reagent: 3% Trichloroacetic Acid/Dichloromethane (RN-1462) or 3% Dichloroacetic Acid/Dichloromethane (RN-1468); (2) Oxidation: 0.1 M iodine in Pyridine/H_2_O/THF (RN-1456) or 0.02 M iodine in pyridine/H_2_O/THF (RN-1455); (3) Cap A: Acetic Anhydride/pyridine/THF (RN-1458); (4) Cap B: 16% N-methylimidazole in THF (RN-7776); (5) Activation reagent: 5-Ethylthiotetrazole 0.25 M in ACN (RN-1466). The LCAA solid supports are also obtained from ChemGenes: *2′-O*-Methyl Uridine 3′-lcaa CPG 1000 Å (N-7904-10, loading capacity 49.8 μmol/g), *2′-O*-Methyl Uridine 3′-lcaa CPG 2000 Å (N-7904-20, loading capacity 31.6 μmol/g), 2′-*O*-Methyl Uridine 3′-lcaa CPG 3000 Å (N-7904-30, loading capacity 24.5 μmol/g), 5′-DMTr 2′-*O*-ALE Uridine 3′-lcaa CPG 2000 Å (N-6384-20, loading capacity 15.8 μmol/g), and 5′-DMTr 2′-*O*-ALE Uridine 3′-lcaa CPG 3000 Å (N-7694-30, loading capacity 20.1 μmol/g). All phosphoramidites were purchased from ChemGenes, including 2′-*O*-ALE, 2′-*O-*TBDMS, and 2′-OMe monomers. (1) ALE monomers: 5′-DMTr-2′-*O*-ALE-Adenosine (N-Lev) 3′-CNEt Phosphoramidite (ANP-7385), 5′-DMTr-2′-*O*-ALE-Guanosine (N-Dmf) 3′-CNEt Phosphoramidite (ANP-7387), 5′-DMTr 2′-*O*-ALE-Cytidine (N-Lev) 3′-Phosphoramidite (ANP-7386), and 5′-DMTr 2′-*O*-ALE-Uridine 3′-Phosphoramidite (ANP-7384); (2) TBDMS monomers: 2′-tBDSilyl Adenosine (N-Bz) 3′-phosphoramidite (ANP-5671), 2′-tBDSilyl Guanosine (N-iBu) 3′-phosphoramidite (ANP-5673), 2′-tBDSilyl Cytidine (N-Ac) 3′-phosphoramidite (ANP-6676), and 2′-tBDSilyl Uridine 3′-phosphoramidite (ANP-5674); and (3) 2′-OMe monomers: 2′-*O*-Methyl Adenosine (N-Bz) 3′-phosphoramidites (ANP-5751). Before synthesis, all phosphoramidites were weighed into clean and dried amber bottles that had been placed under vacuum for >1 h and purged with argon. Phosphoramidites were dissolved in pre-dried ACN immediately before use.

### On MerMade 12 DNA/RNA synthesizer

Oligonucleotides synthesized on a MerMade 12 instrument were carried out on M2-1000 syringe-style columns suitable for 1–10 μmol scale. All amidites/reagents were pressurized under Argon. The synthesis chamber was subjected to a constant flow of 1.5 liter per min during the entire synthesis. Synthesis cycles consisted of (1) detritylation x 3 (each acid treatment delivering 500 µl of reagent in 4 × 8 s pulses; 32 s reaction time); (2) three times of 1 ml DCM wash followed by 1 ml ACN wash; (3) coupling (300 µl activator and 250 µl amidite, 20 s drain time, variable coupling time depending on the monomers); (4) 1 ml ACN wash; (5) capping (250 µl of Cap-A and Cap-B reagents, 30 s reaction time); (6) 1 ml ACN wash; (7) oxidation (700 µl reagent, 18 s reaction time); and (8) 1 ml ACN wash x 2; (9) another capping step was added at the end of each cycle to cap unreacted OH group and react with any residual water that originated from the oxidation solution.

### On K&A S-4-LC or H-8 SE DNA/RNA synthesizer

Oligonucleotides synthesized using K&A instruments (either S-4-LC model or H-8 SE model) were carried out on a 1 μmol scale using a twist-type column with top and bottom frits. The synthesis cycle contained five subroutines: (1) detritylation x 4, each delivered 3 s of the DMTr removal reagent with 10 s wait time, followed by emptying and washing of the synthesis columns with DCM and ACN; (2) coupling, the activator was co-delivered with the amidites for 1 s to the column, followed by another 0.4 s. The coupling time was varied with monomers; (3) capping, both Cap A and Cap B solutions were co-delivered for 2.5 s and 5 s delays, followed by another 1 s delivery and 25 s delays. The optimal capping time was 30 s. Then the column was washed with ACN and purged with argon gas; (4) oxidation, the oxidation solution was delivered for 4 s to the column with the total reaction time of 18 s. Then, the column was washed with ACN and purged with argon gas; (5) another capping subroutine was added at the end of each cycle after oxidation to cap unreacted OH group and react with any residual water that originated from the oxidation solution.

### On-column deprotection of RNA made by ALE monomers

**Scheme 1.**
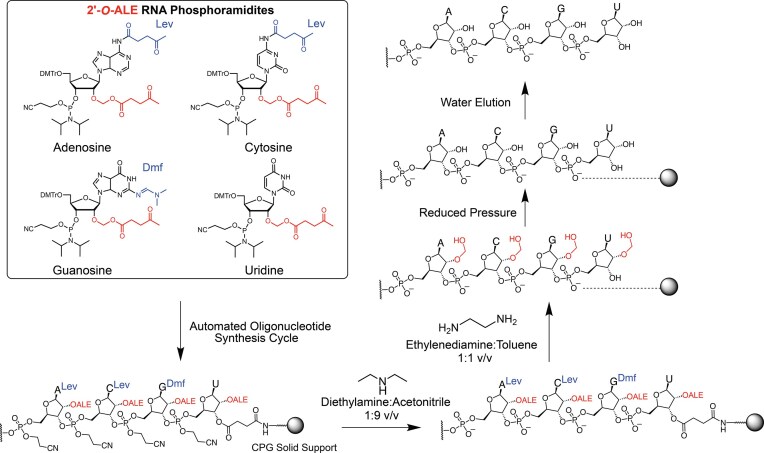
Solid-phase synthesis and on-column deprotection workflow of RNA oligonucleotide synthesized with 2′-acetal levulinic ester (2′-*O*-ALE) phosphoramidites. Following solid-phase assembly, deprotection proceeds with sequential removal of cyanoethyl phosphodiester protecting groups (10% diethylamine/ACN, 20 min), followed by base deprotection and ALE removal via treatment with ethylenediamine/toluene (1:1 v/v) then under reduced pressure to scavenge acetaldehyde byproducts, yielding fully deprotected RNA upon water elution from the solid support.

For RNA synthesized using ALE chemistry, the column was removed from the synthesizer. Whether trityl-on or trityl-off, the deprotection began with decyanoethylation using a 10% diethylamine solution in ACN for 20 min at room temperature. This solution was prepared by adding diethylamine (Fisher Scientific) to anhydrous ACN in a 1:9 v/v ratio. The solution was passed through the column with a 5-ml Luer-lock syringe to deliver fresh solution to the column every 5 min. The column was then washed extensively with 3 × 10 ml anhydrous ACN by syringe. This treatment ensures the removal of backbone cyanoethyl protecting groups, converting the uncharged phosphodiester backbone into its negatively charged form, allowing the freshly synthesized RNA to be retained on the CPG while simultaneously cleaving the chemical anchor (Scheme 1). Then an ethylenediamine (EDA) solution in toluene (1:1 v/v) was prepared by mixing equal volumes of ethylenediamine (Fisher Scientific) and toluene (predried with 3 Å molecular sieves) [21]. The solution was passed through the column with a Luer-lock syringe and subjected to a total of 1 h exposure at room temperature, with fresh solution delivered every 20 min. Then the column was washed extensively with 2 × 10 ml toluene followed by 3 × 10 ml ACN. This reaction was expected to remove all base-protecting groups, the levulinic portion of 2′-ALE, and cleave the succinate ester linker that joins the RNA strand to the support. Next, the column was placed under vacuum for at least 1 h to scavenge the remaining hemiacetal groups. The CPG was then transferred to a 1.5 ml RNase-free microcentrifuge tube, and the crude RNA was eluted by rinsing the support with 4 × 400 μl of RNase/DNase-free water, with the supernatant filtered using a sterile 0.2 μm supor membrane low-binding nonpyrogenic filter (PALL Life Sciences).

### Analytical ion exchange HPLC

The oligonucleotide was analyzed on an analytical HPLC system (Agilent 1200 Series). DNAPac^TM^ PA200 Oligonucleotide HPLC column (8 μm, 4 mm x 250 mm, Thermo Fisher Scientific) was used as the stationary phase, and the mobile phases comprised of 25 mM lithium perchlorate in 25% ACN as buffer A and 0.5 M lithium perchlorate in 25% ACN as buffer B, with constant flow rate of 1 ml/min and column temperature of 65°C. Absorbance signals at λ = 260 nm and 280 nm were monitored. The gradient started with 100% buffer A for 2 min, followed by 0%–30% buffer B from 2 min to 30 min. The column was washed with 100% buffer B for 10 min followed by equilibration with buffer A for 10 min before the next injection.

### RNA purification via DMTr-ON ion-pairing reverse phase HPLC

For DMTr-ON or CPR-II tagged long RNA purification, the crude samples were filtered through 0.22 µm and purified by reverse-phase HPLC using an Agilent 1260 Infinity II preparative system equipped with a C18 column (Agilent Prep 100 Å C18, 50 × 50 mm, 5 µm) maintained at 60°C. The mobile phases consisted of Buffer A (0.1 M triethylamine acetate (TEAA), pH 7.0) and buffer B (HPLC grade ACN). The gradient went from 10% to 35% of buffer B over 20 min at a flow rate of 5 ml/min. Absorbance signals at λ = 260 nm and 280 nm were monitored. The second major peak, which contains the DMTr-ON full-length product, was collected and recovered by alcohol precipitation.

C19-nitrobenzyl-tagged minimal mRNAs were purified using the same HPLC system and stationary phase, with modified gradient and mobile phases. Buffer A consisted of 50 mM TEAA (pH 7.0), and buffer B was HPLC-grade ACN. The separation gradient began with 5% buffer B for 3 min, followed by a linear increase from 15% to 50% buffer B over 20 min at a flow rate of 5 ml/min. The second major peak, which contains the C19-nitrobenzyl-tagged full-length product, was collected and recovered by alcohol precipitation.

### Oligonucleotide quantification

Oligonucleotide was quantified using Nanodrop spectrophotometer (DeNovix DS-11 Series Spectrophotometer) by applying 2 μl of the sample and water as the blank. Absorbance at λ = 260 nm was measured, and the molar extinction coefficient from the nearest-neighbor method was used to calculate the final yield.

### Oligonucleotide ESI-LC-MS

Oligonucleotides were analyzed by LC‐MS using a Dionex Ultimate 3000 UHPLC coupled to a Bruker Maxis Impact QTOF in negative ESI mode. Samples were run through a Phenomenex Luna C18(2)‐HST column (2.5 μM, 120 Å, 2.1 × 100 mm) using a gradient of 90% mobile phase A (100 mM HFIP, 5 mM NEt_3_ in H_2_O) and 10% mobile phase B (MeOH) to 40% mobile phase A and 60% mobile phase B over 20 min. The data was processed, and spectra were deconvoluted using the Bruker DataAnalysis software version 4.2.

### *In vitro* transcription of gRNA

gRNA was *in vitro* transcribed in T7 buffer (40 mM Tris buffer pH 7.9, 2.5 mM spermidine, 26 mM MgCl_2_, 0.01% Triton X-100), NTPs (8 mM GTP, 5 mM CTP, 5 mM ATP, 2 mM UTP), 10 mM DTT, 1 U/μl T7 RNA polymerase and 2X PCR generated DNA templates carrying a T7 promoter sequence. The mixture was incubated for 2 h at 37°C. The reaction was stopped by adding denaturing loading dye (49% formamide, 0.5 mM EDTA, 0.0125% xylene cyanol, and 0.0125% bromophenol blue) followed by heating to 95°C. The transcription products were run on 8% polyacrylamide gel electrophoresis (PAGE). RNA bands were visualized by UV shadowing, cut out, and left to elute overnight in 300 mM NaCl at 4°C on a rotator. The RNA was then ethanol precipitated, the pellet was resuspended in H_2_O, and stored at –20°C. RNA concentrations were determined by measuring absorbance at 260 nm in H_2_O on a Nanodrop. Molar extinction coefficients were calculated using the nearest neighbor method.

### Cas9 cleavage assay

The dsDNA substrate was generated by annealing two oligonucleotides purchased from Integrated DNA Technologies (IDT). The complementary strand was radiolabeled using T4 polynucleotide kinase (New England Biolabs) and [γ-^32^P]-ATP (Revvity) in 1X T4 polynucleotide kinase reaction buffer at 37°C for 30 min. After heat inactivation (65°C for 20 min), denaturing loading dye was added to the samples, followed by heating to 95°C for 5 min. The oligonucleotides were then purified via PAGE, eluted, and concentrated as mentioned before. Duplex substrates (100 nM) were generated by annealing the radiolabeled complementary oligonucleotide with equimolar amounts of unlabeled noncomplementary oligonucleotide at 95°C for 5 min and were slowly cooled to room temperature. Cas9-mNeonGreen (50 nM final concentration) was preincubated for 15 min at room temperature with gRNA (100 nM final concentration) in buffer (50 mM Tris pH 7.5, 100 mM KCl, 10 mM MgCl_2_, 100 μg/ml Recombinant Albumin (NEB)) in a total volume of 76 μl. Reactions were initiated by the addition of 4 μl target DNA (5 nM final concentration) and incubated at 37°C. 10 μl was taken out at each timepoint (30 s, 1 min, 2 min, 4 min, 8 min, 16 min, and 32 min) and added immediately to 10 μl of denaturing loading dye to quench the reaction. Samples were heated to 95°C for 5 min, and the cleavage products were resolved on 8% PAGE and were visualized by phosphorimager.

### Fluorescence binding assay with TO1-B ligand

gRNA (100 nM final) was incubated in buffer (50 mM Tris pH 7.5, 100 mM KCl, 1 mM MgCl_2_, 100 μg/ml Recombinant Albumin (New England Biolabs)) containing 50 nM TO1-B (Applied Biological Materials) for 1 h at room temperature before reading fluorescence emission. All fluorescent measurements were taken using a Varian Cary Eclipse spectrophotometer; samples were excited at 510 nm, and emission values were recorded at 535 nm.

### Cell-based editing via EGFP knockout

HEK293T cells stably expressing EGFP and SpCas9-NLS were a gift from the Wen Xue laboratory (UMass Medical Center). Cells were maintained at 37°C and 5% CO_2_ in 1 × minimum essential medium (MEM, Corning) supplemented with 5% fetal bovine serum (FBS, Gibco), 5% cosmic calf serum (CCS, Gibco), and 1% nonessential amino acids (NEAA, Gibco). Cell-based gene editing was performed as previously reported [28, 29]. Briefly, transfections were carried out with Lipofectamine RNAiMAX (Thermo Fisher Scientific) according to the manufacturer’s instructions with minor modifications. For each well of a 96-well plate, 5 pmol sgRNA targeting EGFP was mixed with RNAiMAX in Opti-MEM (Gibco) and incubated for 20 min at room temperature. Subsequently, 40,000 cells diluted in Opti-MEM were added to each well to a final volume of 200 µl. Cells were incubated for 8–10 h at 37°C, after which an equal volume of full-serum medium (5% FBS, 5% CCS, and 1% NEAA) was added to each well and incubated for an additional 16 h. Following the incubation, the medium was replaced with fresh full-serum medium, and cells were grown until 120 h posttransfection. At 120 h (5 days), EGFP expression was quantified by flow cytometry. Cells were washed with 1 × PBS (Gibco), trypsinized, and centrifuged at 500 ×*g* for 5 min. The cell pellet was washed with 1× PBS, centrifuged again, resuspended in 1 × PBS, and transferred to a new tube. Resuspended cells were analyzed on a BD LSRFortessa X-20 (BD Biosciences). Forward and side scatter were used for doublet discrimination. EGFP fluorescence was measured using a 488 nm laser for EGFP excitation. Data were acquired with FACSDiva (BD FACSDiva), and analysis was performed in FCS Express (Dotmatics). Gates were set using untreated controls run in parallel.

### *In vitro* mRNA translation

The *in vitro* translation was carried out in a Krebs-II cell-free extract as described [30, 31]. In brief, a 10 µl reaction contained 5 μl of Krebs-II extract and the following components: 75 mM HEPES-KOH (pH 7.3), 25 mM KCl, 100 mM KOAc, 5.25 mM MgCl_2_, 2 mM DTT, 0.25 mM spermidine, 1 mM ATP, 0.2 mM GTP, 0.2 mM CTP, 0.2 mM UTP, 10 mM creatine phosphate, 80 μg/ml creatine kinase, and 0.04 mM amino acids with 20-300 ng RNA. The reaction mixture was incubated at 37°C for 1 h. The translation mixture was directly mixed with an equal volume of Nano-Glo HiBiT Lytic buffer prepared from Nano-Glo HiBiT Lytic Detection System (Promega). The mixture was transferred to a white 96-well plate (Corning). The luminescence was then read on a BioTek Synergy H1 multimode reader (Agilent) with an integration time of 5 s.

### Cellular mRNA translation

HeLa or HEK293 cells were cultured at 37°C with 5% CO_2_ in Dulbecco’s Modified Eagle Medium, high glucose (DMEM, Gibco) supplemented with 10% fetal bovine serum (FBS, Gibco), 100 U/ml Penicillin, and 100 µg/ml Streptomycin (Gibco). Before transfection, cells were seeded at 8 × 10^3^ cells per well in 96-well plates with 100 μl DMEM and incubated overnight to achieve confluence. Cells were transfected with 200 ng RNA per well using 0.3 μl Lipofectamine™ MessengerMAX™ Transfection Reagent (Invitrogen) in a total volume of 100 μl Opti-MEM I reduced serum medium (Gibco). After 4 h of incubation, the Opti-MEM medium was replaced with supplemented DMEM, and cells were incubated overnight. At 24 h posttransfection, cell culture medium was removed, and wells were washed twice with 100 μl PBS. HiBiT expression was quantified using the Nano-Glo HiBiT Lytic Detection System (Promega). Cells were lyzed with 20 μl Nano-Glo HiBiT Lytic buffer prepared according to the manufacturer’s protocol and incubated for 10 min at room temperature. Cell lysates were diluted with 50 μl PBS and transferred to white 96-well plates (Corning) at 50 μl per well. Luminescence was measured using a BioTek Synergy H1 multimode reader (Agilent) with an integration time of 5 s.

### MazF digestion of minimal mRNA

0.1 nmol of the 5′-monophosphorylated RNA was digested with mRNA interferase MazF enzyme (TaKaRa). The 85 μl reaction mixture contains 0.1 nmol of RNA substrate, 10 μl of MazF enzyme (20 U/μl), and 17 μl of 5× MazF Buffer. The reaction mixture was incubated at 37°C for 2 h. The mixture was then cleaned up with Monarch^®^ Spin RNA Cleanup Kit (New England Biolabs) and eluted with 10 μl of DNase/RNase-free water. The eluted RNA fragment mixture was subjected to LC-ESI-HRMS analysis directly.

## Results

### Solid-phase synthesis

Our first syntheses were carried out to evaluate the suitability of using ALE monomers for routine long RNA synthesis and their potential advantages over conventional TBDMS chemistry. A sgRNA (*AK99*) designed to target the enhanced green fluorescent protein (*EGFP*) reporter gene was prepared under various conditions. Syntheses were conducted on an automated MerMade 12 synthesizer starting from 1 µmol of a succinyl-linked 2′-OMe-rU-CPG solid support (2000 Å pore size). ALE and TBDMS monomers were dissolved in acetonitrile (ACN) at concentrations of 50 mM (ALE and TBDMS) or 100 mM (TBDMS) and allowed to couple in the presence of 5-ethylthio-tetrazole activator (ETT; 0.5 M in ACN). Coupling times for ALE monomers were arbitrarily set at 2 min for A/C/U and 3 min for G, and 2-8 min for TBDMS monomers. The other steps in the cycle were kept constant and consisted of capping (Ac_2_O/16% NMeIm/THF/py; 30 s); oxidation (0.1M I_2_ in py/water; 18 s); and detritylation (3% trichloroacetic acid in dichloromethane (DCM); 3 × 30 s). The final DMTr group was removed on the CPG in all cases.

**Figure 2.**
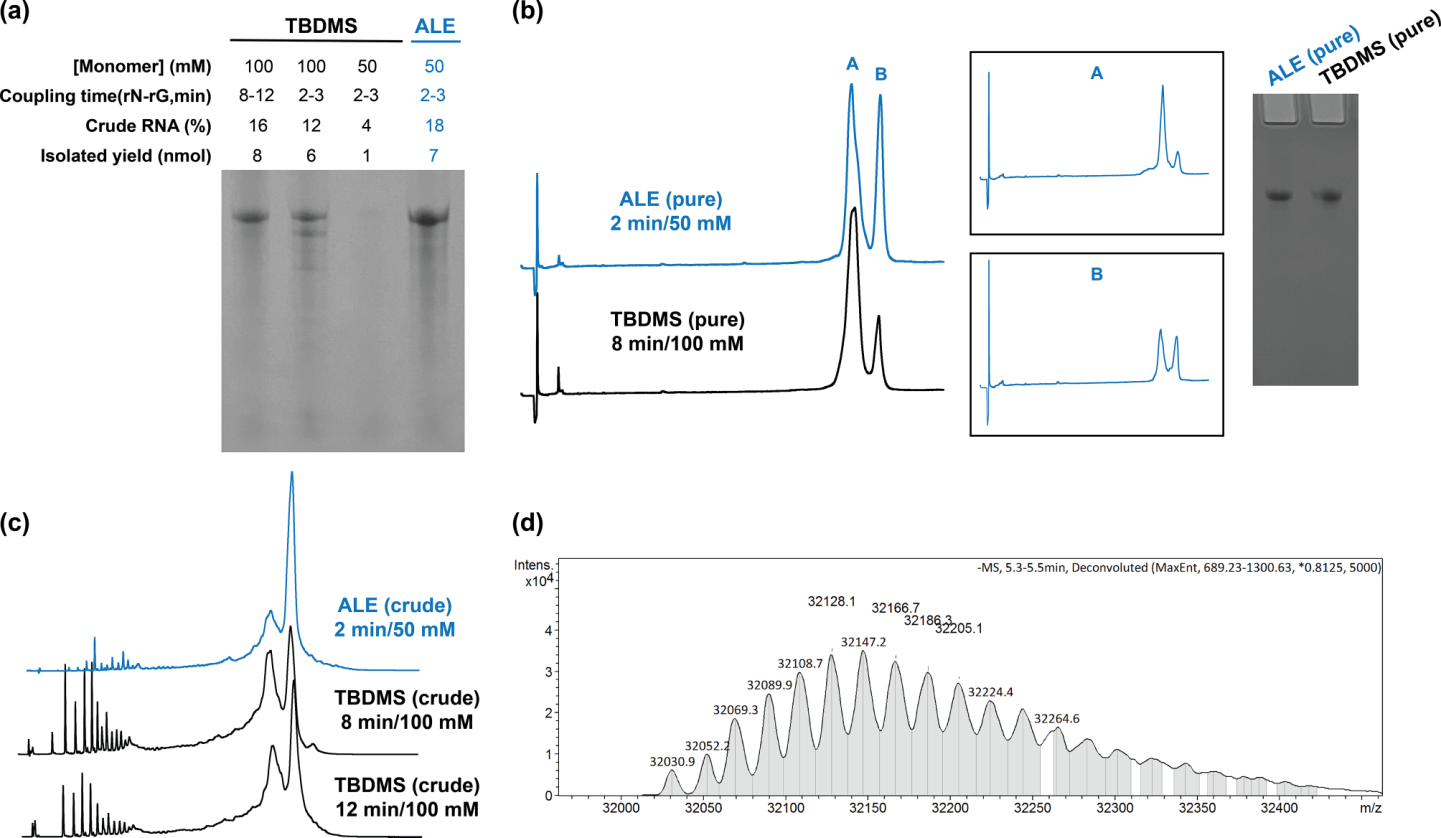
Preliminary synthesis of a 99-mer sgRNA *AK99* sequence. (**a**) 10% denaturing PAGE gel analysis of crude synthesis using 2′-O-ALE chemistry compared with the 2′-OTBDMS chemistry under different coupling conditions. (**b**) Ion-exchange chromatogram and 10% denaturing PAGE gel analysis of the purified samples from the PAGE gel, which shows double peaks due to secondary structure of sgRNA. Peaks A and B were collected individually and re-injected, again showing two close moving peaks. (**c**) Ion-exchange chromatogram of crude samples synthesized under different conditions. (**d**) Deconvoluted ESI-LC-MS spectrum of the purified product. Theoretical MW: 32030.0 Da. Found MW: 32030.9 Da.

The gRNA prepared via ALE chemistry was deprotected by first pushing through the column with a solution of diethylamine in ACN (1:9 v/v, r.t., 20 min) to cleave the phosphate protecting groups (Scheme 1). Additional ACN was passed through the column to remove any acrylonitrile byproduct remaining. Next, the CPG was treated with dry ethylenediamine (EDA)/toluene solution (1:1 v/v) over a 2-h period to simultaneously cleave the levulinyl (Lev) groups on adenine (N^6^) and cytosine (N^4^), the dimethylformamidine (Dmf) group on guanine (N^2^), as well as the 2′-ALE groups and succinyl linker [21]. Because phosphate bridges are charged at this stage, the RNA remains physically adhered to the CPG in the nonpolar and aprotic toluene, allowing subsequent washes with toluene and ACN to remove impurities. After vacuum drying (1 h), the crude RNA was eluted by washing the support with DNase/RNase-free water. RNA strands prepared via TBDMS chemistry were deprotected with conc. NH_4_OH/ethanol (3:1 v/v, 48 h, r.t.) followed by treatment with anhydrous N-methylpyrrolidinone (NMP)/triethylamine(TEA)/triethylamine trihydrofluoride (TREAT-HF) (3:4:6 v/v, 2.5 h, 65°C) and recovered by butanol precipitation. The results from these preliminary trials are shown in Fig. 2a. A fraction (1/4) of the 1 µmol scale crude products was purified by denaturing polyacrylamide gel electrophoresis (PAGE), and the recovery from this fraction was translated into the isolated yield for 1 µmol synthesis. The data showed that when the concentrations and coupling times of ALE and TBDMS monomers were both kept at 50 mM and 2 min, respectively, ALE chemistry yielded more product (PAGE purification). However, ALE and TBDMS chemistries performed similarly when the concentration of TBDMS monomers was doubled (100 mM) and the coupling time quadrupled (8 min) yielding ca. 7-8 nmol of RNA recovered after extraction from the gel. Furthermore, the sgRNAs produced by ALE chemistry were comparable to RNA produced by TBDMS-protected monomers when evaluated by ion-exchange HPLC (IEX-HPLC) (Fig. 2b). Notably, the crude ALE samples showed fewer early-eluting failure products compared to TBDMS syntheses (Fig. 2c). Interestingly, we observed that the purified sgRNA *AK99* occasionally displayed two well-resolved peaks in variable ratios between injections. When the individual peaks A and B were collected and re-injected, the double-peak pattern reappeared. We attribute this observation to different RNA secondary structures or conformers rather than impurities, as the analysis was performed under non- or mildly denaturing IEX-HPLC conditions (pH 7.0, 60°C), consistent with previously reported observations [32]. Both the denaturing PAGE gel in Fig. 2b and LC-MS characterization in Fig. 2d further validated the presence of homogenous full-length product and the purity of the synthesized sgRNA.

### ALE synthesis optimization

**Figure 3.**
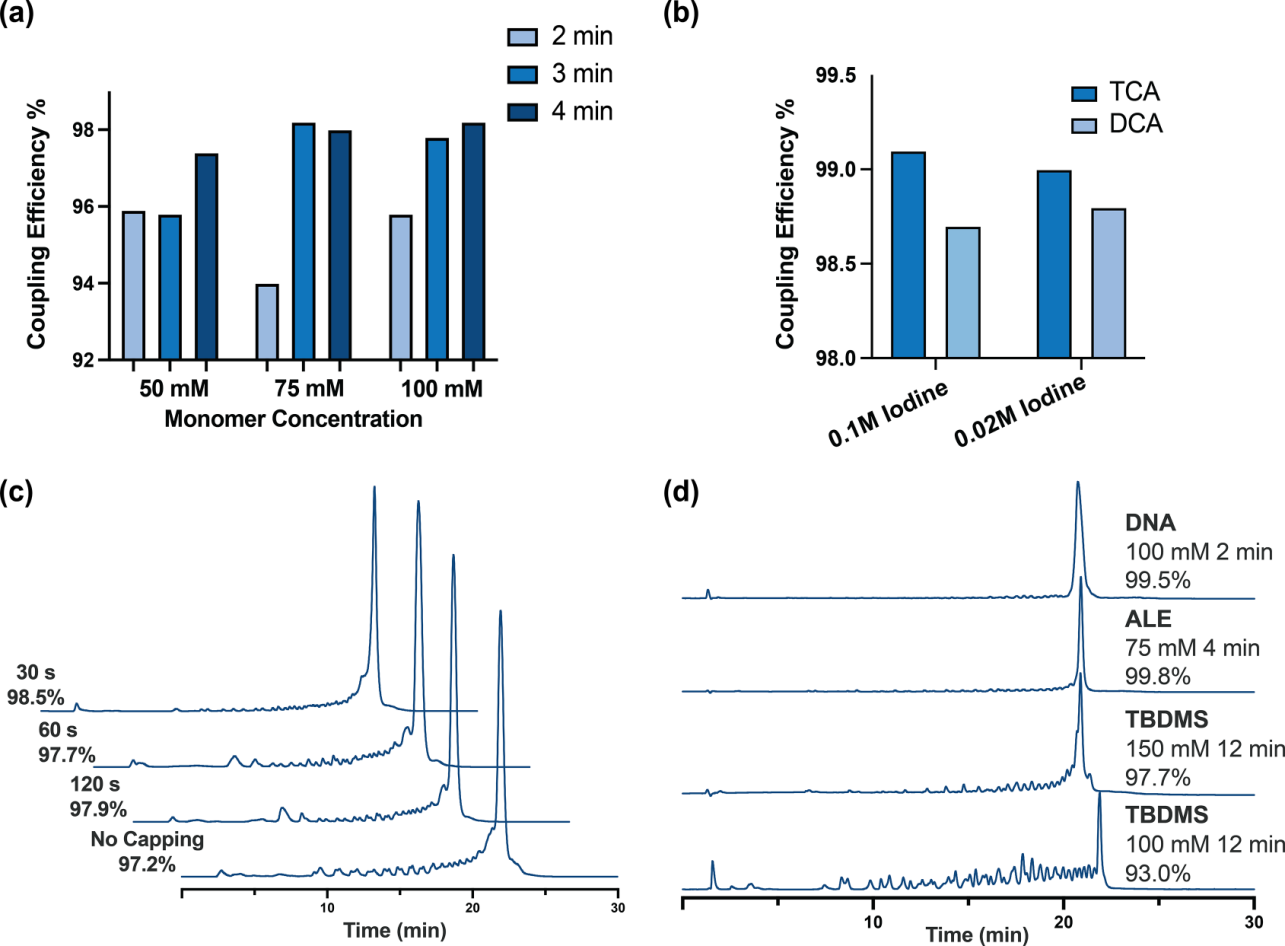
Optimization of solid-phase synthesis using 2′-*O*-ALE phosphoramidites with 26-mer RNA *AK26*. (**a**) Coupling efficiency of the synthesis with different monomer concentrations, 50, 75, and 100 mM under 2–4 min coupling time. (**b**) Coupling efficiency of the synthesis with different oxidation and detritylation reagents. (**c**) Ion-exchange HPLC chromatograms of the *AK26* sequence made under different capping time (30, 60, 120 s, and no capping) after coupling step. (**d**) Ion-exchange HPLC chromatograms of the crude *AK26* synthesis synthesized with ALE under optimized conditions, compared with TBDMS and DNA monomers.

Encouraged by the preliminary *AK99* synthesis, we then moved to fully optimize our procedures. We undertook the synthesis of a short 26-nt long RNA of mixed base composition (*AK26*). Again, syntheses were carried out on a MerMade 12 synthesizer, using the synthesis cycle described above for *AK99*. ALE monomers were coupled at various concentrations (50, 75, and 100 mM) and coupling times (2, 3, and 4 min). We found that ALE amidites coupled more efficiently at 75 mM compared to 50 mM, and that no additional improvement was observed if the amidite concentration was increased to 100 mM (Fig. 3a; Supplementary Fig. S1a and b). We also found that a 3–4 min coupling time resulted in higher coupling efficiency compared to a 2-min coupling time previously used in the synthesis of *AK99* (Fig. 2). Additionally, we observed that the rG-ALE amidite did not require an extended coupling time as we normally apply to rG-TBDMS monomers (Supplementary Fig. S1c). Further variations in capping time, oxidation, and detritylation conditions did not lead to improvements (Fig. 3b and c; Supplementary Fig. S1d). Under these optimized conditions, ALE monomers coupled with the same efficiency as DNA monomers (ca. 99.5%) (Fig. 3d).

### Deprotection with EDA/toluene

**Figure 4.**
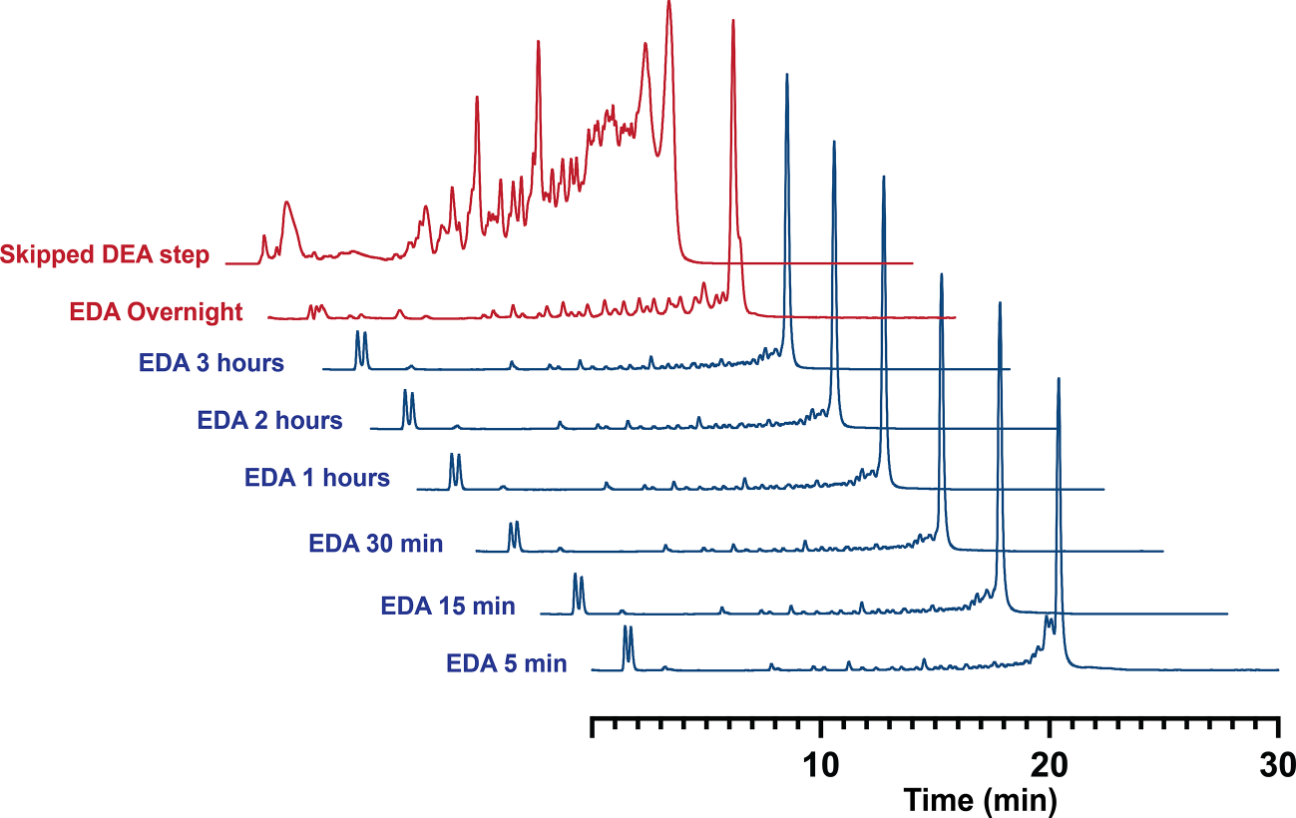
Time-course analysis of EDA/toluene deprotection for ALE-made RNA (*AK26*). Ion-exchange HPLC chromatograms monitoring deprotected crude product with EDA treatment of 5 min to 3 h, following universal initial cyanoethyl removal with diethylamine for 20 min. Extended treatment (overnight) resulted in RNA degradation, while skipping the cyanoethyl removal step (top trace) causes severe pre-mature inter-nucleotide cleavage.

The deprotection of the protected oligoribonucleotide must avoid chain cleavage and/or isomerization of the phosphodiester linkages. Literature precedent demonstrates that under anhydrous nonpolar solvents such as toluene, deprotonation of the 2′-OH group under basic conditions (affording a more reactive alkoxide) is unfavoured, thereby preventing strand cleavage via intramolecular attack on phosphodiester bonds [21]. To investigate this further, we monitored the ethylenediamine (EDA)-based deprotection of RNA *AK26* using IEX-HPLC over several hours (Fig. 4). First, cyanoethyl phosphate groups were removed by treatment with 10% diethylamine (DEA) in ACN at room temperature for 20 min, generating a more stable phosphodiester backbone. We found the elimination of this step resulted in significant RNA degradation as shown in Fig. 4. Following this, the support was rinsed with ACN and treated with a mixture of EDA and toluene (1:1 v/v) at room temperature. Small portions of the support were removed from this solution over a 16-h period, then rinsed with ACN and finally washed with water to release the crude RNA product. HPLC and LC-MS analysis revealed that under these conditions, EDA/toluene effectively removes protecting groups from nucleobases within 30 minutes (Supplementary Fig. S2). Prolonged treatment (3 h) did not alter the integrity of the sample, as confirmed by the similarity of its chromatogram. However, since the overnight treatment with EDA resulted in noticeable degradation, we concluded that 1-h exposure is sufficient and optimal for complete deprotection while minimizing RNA degradation. The EDA deprotection process can be extended as needed to efficiently remove less labile protecting groups on exocyclic amines, such as benzoyl (Bz), without causing RNA degradation. The use of Unylinker supports or N-iBu-protected guanosines is discouraged because their quantitative cleavage requires heat.

### Optimized synthesis of single-guide RNAs (sgRNAs)

**Figure 5.**
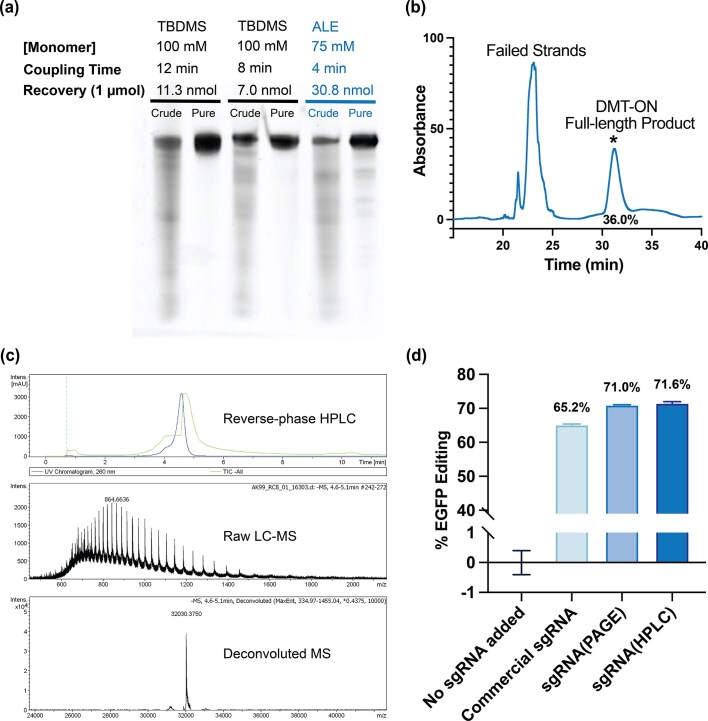
Chemical synthesis and functional validation of sgRNA (*AK99*) using optimized ALE and TBDMS conditions. (**a**) 10% denaturing PAGE analysis showing ALE chemistry (75 mM, 4 min coupling) achieved higher recovery compared to TBDMS chemistry (100 mM, 8 or 12 min). (**b**) DMTr-ON RP-HPLC purification of ALE-synthesized AK99 showing a full-length product of 36.0%. (**c**) High-resolution LC-MS characterization. Reverse-phase HPLC trace (top), raw mass spectrum (middle), and deconvoluted spectrum (bottom). Theoretical MW: 32030.0403 Da. Found MW: 32030.3750 Da. (**d**) % EGFP editing of HEK293T cells followed by blast transfection of the commercially sourced and either PAGE- or HPLC-purified ALE-synthesized *AK99* sgRNA.

Having established the optimal parameters for the efficient synthesis of RNA strands with ALE chemistry, we resynthesized sgRNA *AK99* on a 1 µmol scale via ALE and TBDMS chemistry, this time using a K&A synthesizer from Sierra Biosystems to ensure reproducibility across different instruments. The synthesis cycles and deprotection conditions used are summarized in Table 1, with complete experimental details provided in the Materials and Methods section. Each deprotected oligomer was initially analyzed and purified by denaturing PAGE (Fig. 5a), where the full-length product was excised, extracted with a saline solution, and desalted by size-exclusion chromatography. The yield of product prepared from ALE monomers was superior despite the increased concentration and extended coupling times used for TBDMS monomers. The overall yields of *AK99* via ALE syntheses ranged from 22 to 31 nmol (2.2%–3.1%), compared to 7 to 11 nmol (0.7%–1.1%) for TBDMS synthesis.

**Table 1.** Comparison of key experimental parameters of TBDMS versus ALE-based synthesis. ALE chemistry enables milder deprotection conditions, reduced reaction times, and on-support processing

	TBDMS	ALE
**[Monomer]**	100 mM	75 mM
**Coupling time**	8–12 min	4 min
**Deprotection**		
a. Phosphate		Et_2_NH/ACN, 20 min
b. Nucleobase	NH_4_OH/EtOH, 55°C, 16 h	EDA/Toluene, 1 h
c. 2′-deprotection	Fluoride, 1.5 h	Reduced pressure, 1 h
**Status**	RNA in solution	RNA on support
**Isolation**	Alcohol precipitation	Elution with H_2_O
**Purification**	HPLC or PAGE Gel	HPLC or PAGE Gel

While denaturing polyacrylamide gel electrophoresis is a widely used and cost-effective method for RNA purification, it is labor-intensive and presents challenges with longer RNA molecules as product recovery and band resolution decrease as the RNA’s length increases. This led us to consider reverse-phase HPLC purification, leveraging the hydrophobic 5′-DMTr protecting group to differentiate full-length oligonucleotides from truncated failure sequences [33, 34]. A critical advantage of the ALE deprotection protocol is its compatibility with DMTr-ON purification, where the mild basic EDA/toluene conditions successfully retain the 5′-DMTr group, avoiding acidic TREAT-HF reagent or elevated temperatures that typically cause DMTr group loss. By carefully optimizing the choice of column and elution gradient, we achieved effective separation of full-length product from truncated sequences for this 99-nt sgRNA, as shown in Fig. 5b. An average coupling efficiency of 99% was estimated by analyzing the percentage area of the product peak (36%) from the HPLC chromatogram. The identity and purity of the purified product were further confirmed by high-resolution LC-MS analysis (Fig. 5c).

To evaluate gene-editing activity, we transfected HEK293T cells with either the synthetic sgRNA *(AK99*) or a commercially sourced sgRNA targeting EGFP. Our results showed that *AK99* achieved over 70% EGFP knockout efficiency, significantly outperforming the commercial control irrespective of the purification method used (Fig. 5d).

### Chemical synthesis of fluorescently tagged RNA complexes

**Figure 6.**
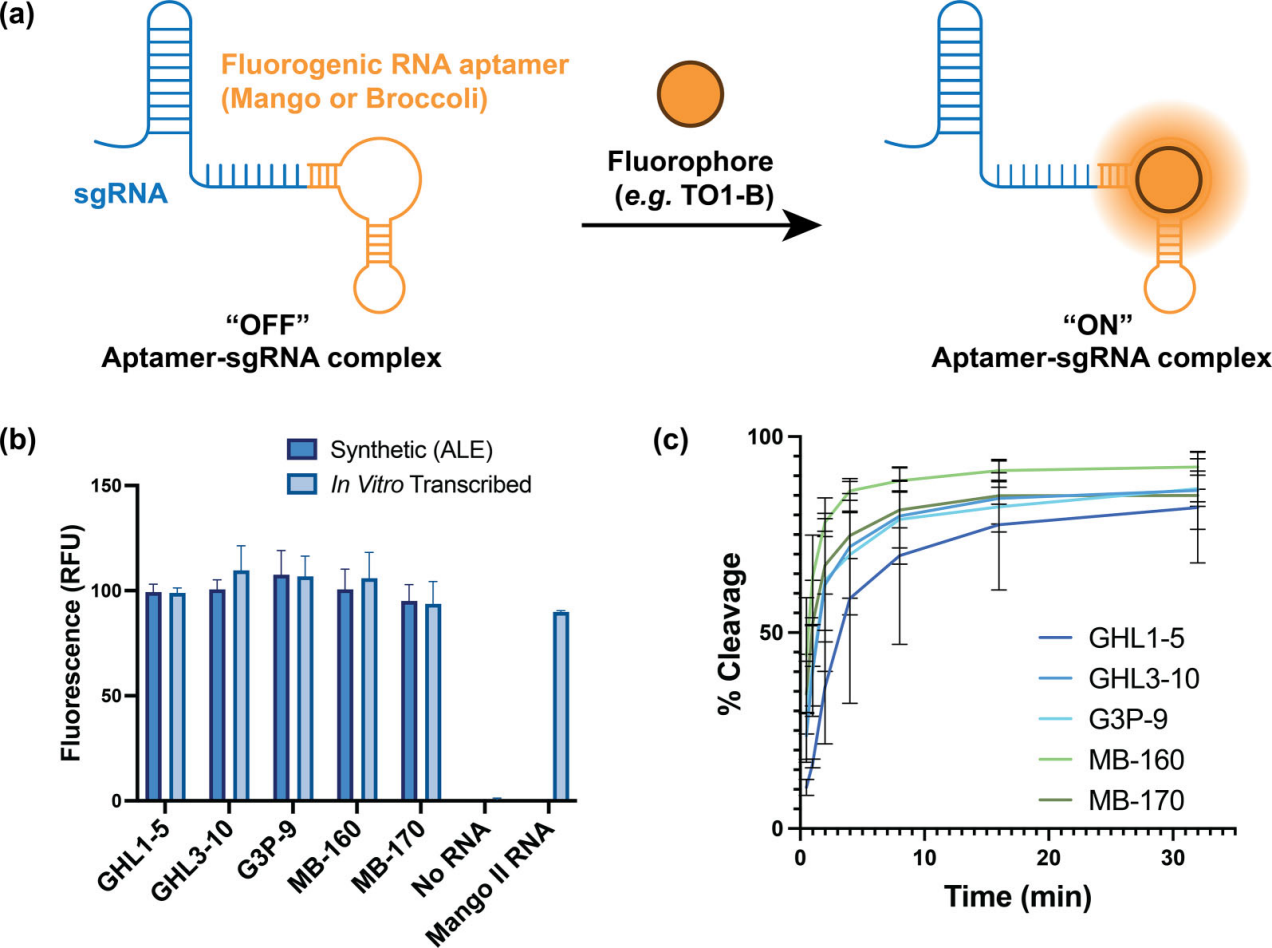
Chemical synthesis and functional validation of fluorogenic sgRNA complexes tagged with Mango II and Broccoli aptamers. (**a**) Schematic illustration of the Mango II aptamer-TO1-biotin system. The TO1-biotin fluorophore exhibits enhanced fluorescence upon specific binding to Mango II RNA aptamers, enabling RNA visualization and tracking. (**b**) Fluorescence binding assays with TO1-B ligand.100 nM of the Mango II-tagged sgRNA was incubated with 50 nM TO1-B at room temperature for 1 h before reading (*Ex*. 510 nm, *Em*. 535 nm). *In vitro* transcribed Mango II RNA aptamer was used as a positive control and no RNA addition was used for a negative control. (**c**) *In vitro* Cas9 cleavage assays of the ALE-synthesized fluorogenic sgRNAs against a 52-bp dsDNA target.

Mango II or Broccoli aptamers, when conjugated to RNA strands, act as bright, high-affinity fluorogenic probes, enabling the visualization and tracking of specific RNA molecules *in vitro* and in living cells [35–39]. These aptamers undergo dramatic fluorescence enhancement upon binding their cognate ligands (*e.g*. TO1-Biotin) as shown in Fig. 6a.

To construct more complex and longer RNA, we used ALE chemistry to design fluorogenic 109-133 nt sgRNAs (*GHL1-5, GHL3-10*, and *G3P-9*) incorporating Mango II aptamers that target a 52 base-pair double-stranded DNA sequence (Supplementary Fig. S3; Supplementary Table S2). Following syntheses, the RNA strands were analyzed and purified by PAGE (Supplementary Fig. S4a) and characterized by high-resolution LC-MS (Supplementary Fig. S5–S7). Consistent with previous results, ALE monomers yielded superior product recovery compared to TBDMS chemistry (Supplementary Table S3). Next, we synthesized longer sgRNA constructs incorporating both Broccoli and Mango II aptamers [*MB160* (160-nt) and *MB170* (170-nt)]. Purity was again assessed via PAGE gel in Supplementary Fig. S4b. Using a larger pore size (3000 Å) CPG for these longer RNAs significantly improved both yield and purity (Supplementary Table S4; Supplementary Fig. S4c). To test the synthetic RNAs, we performed fluorescence binding assays with TO1-B. As shown in Figure 6b, all sgRNAs were functional and showed enhanced ligand fluorescence regardless of aptamer position. Furthermore, *in vitro* cleavage assays (Fig. 6c) showed that all tagged sgRNA retained robust Cas9-mediated cleavage activity against the 52-bp dsDNA target, with over 80% cleavage at the endpoint. These results demonstrate that ALE chemistry can produce long, highly structured, and functional fluorogenic RNAs exceeding 150 nucleotides in length.

### Chemical synthesis of native and modified translatable minimal mRNA

Successful mRNA vaccines for COVID-19 have established mRNA technology as a transformative platform for various therapeutic applications, such as personalized cancer immunotherapy [40–44]. Current mRNA production predominantly relies on IVT, which is suited for long RNA sequences encoding the full-length protein. However, IVT processes face several challenges related to the sourcing of reliable polymerases, the potential for sequence errors, and limitations in precisely incorporating chemical modifications. Additionally, T7 RNA polymerase-based IVT can extend premature termination products through its RNA-dependent RNA polymerase activity, generating immunostimulatory double-stranded RNA by-products [45, 46].

Chemical synthesis of native or modified RNA fragments, subsequently ligated together, offers an attractive strategy to achieve site-specific chemical modification in mRNA [47], gaining particular importance with the rise of minimalist mRNA cancer vaccines as next-generation therapeutic candidates [41, 42]. By encoding only essential elements for short neoantigens (8–25 amino acids), synthetic minimal mRNA can provide enhanced specificity, improved stability, and simpler manufacturing than traditional IVT-mRNA platforms. Achieving robust access to synthetic RNA fragments exceeding 200 nucleotides would enable broader implementation of these precision approaches.

**Figure 7.**
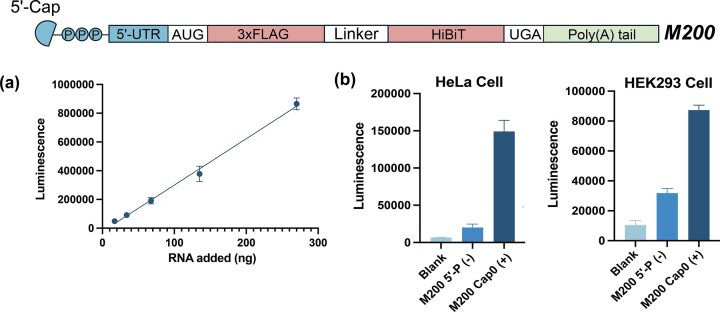
Chemical synthesis and functional validation of minimal mRNA (*M200*). The *M200* construct contains a 5′-cap, 5′-UTR, coding sequence for 3 × FLAG and HiBiT peptides connected by a peptide linker, and poly(A) tail. (**a**) Dose-dependent luminescence activity of ALE-synthesized *M200* in the Krebs-II cell-free translation system. (**b**) Cellular translation in HeLa (left) and HEK293 (right) cells comparing uncapped 5′-phosphorylated (-) versus 5′-capped (+) *M200*, showing three- to sevenfold enhancement in protein expression with capping. HiBiT expression was measured 24 h posttransfection in triplicate from 96-well plates.

Toward this goal, we first attempted the synthesis of a 200-mer minimal mRNA (*M200*) encoding two epitope peptides, 3 × FLAG and HiBiT via ALE chemistry (Fig. 7). The mini-mRNA consisted of a 52-nt 5′-untranslated region (5′-UTR), a 123-nt coding sequence, and a 25-nt poly(A) tail. The translational efficiency of this construct can be quantified by measuring luminescence produced by the HiBiT tag [48, 49]. Solid-phase synthesis (1 µmol; 3000 Å CPG) was completed by coupling the hydrophobic 5′-phosphorylation reagent II (CPR II) developed by Löhnberg, which enables the subsequent DMTr-ON ion-pairing reverse-phase HPLC (IP-RP HPLC) analysis and purification (Supplementary Scheme S1) [50]. The isolated yield of *M200* was 7 nmol, with purity assessed by 6% denaturing PAGE gel (Supplementary Fig. S8a). Analysis of the IP-RP HPLC chromatogram revealed a product peak comprising 27.4% of the total area, corresponding to an average monomer coupling efficiency of 99.4% (Supplementary Fig. S8b).

To install the 5′ cap on the minimal mRNA, we adopted the method of Abe and coworkers [49], where a 5′-monophosphorylated RNA is reacted with N^7^-methylated GDP imidazolide (Im-m^7^GDP) in the presence of 1-methylimidazole in DMSO (Supplementary Scheme S2). Although PAGE could not resolve the capped from the uncapped mRNAs with 200 nucleotides, these conditions resulted in ~80% capping efficiency when we applied them to a shorter, model sequence *AK26* (Supplementary Fig. S9).

Next, we evaluated the translational efficiency of 5′ capped *M200 in vitro* (Krebs-II cell-free translation system) and in both cultured HeLa and HEK293 cells. The Krebs II system simplifies translation studies by providing all the necessary molecular machinery for protein synthesis outside the complex environment of a cell [31]. Under these conditions, 5′-capped *M200* showed a dose-dependent luminescence signal, indicative of translation of this mRNA *in vitro* (Fig. 7a), with a three- to sevenfold higher efficiency than uncapped 5′-p M200 (-) mRNA in cultured cells (Fig. 7b).

**Figure 8.**
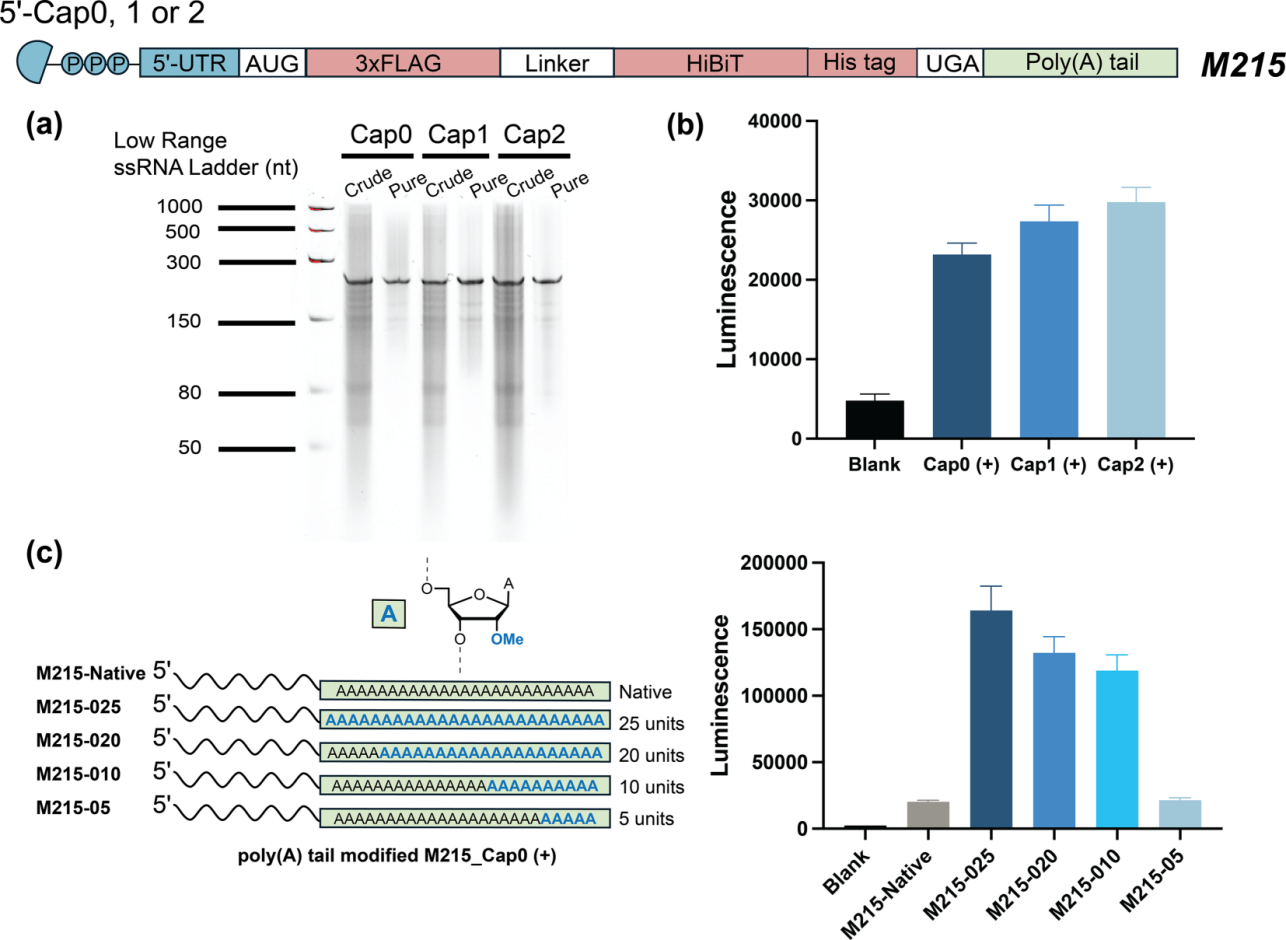
Chemical synthesis and functional validation of the 215-nt minimal mRNA (*M215*). (**a**) 6% denaturing PAGE analysis of Cap_0, Cap_1, and Cap_2 *M215* constructs before (crude) and after purification. (**b**) HiBiT expression from the various 5′-capped (+) *M215* constructs was measured in 96-well plates cultured HeLa cells after 24 h of transfection with lipofectamine. (**c**) Introduction of chemical modification at the poly(A) tail of Cap_0 *M215*, with various numbers (25, 20, 10, and 5 units) of 2′-OMe nucleotides. HiBiT expression was measured in 96-well plates of cultured HeLa cells after 24 h of transfection.

Based on these encouraging results, we extended the RNA length beyond 200 nucleotides by incorporating an additional His-tag, yielding a 215-nucleotide mRNA (*M215*). To our knowledge, this represents the longest chemically synthesized RNA to date. Three *M215* mRNA variants were synthesized to include one of three distinct cap structures: Cap_0 (*m*^7^*GpppN*), Cap_1 *(m*^7^*GpppN*_m_), and Cap_2 (*m*^7^*GpppN*_m_*N*_m_). These cap structures were readily incorporated into the mRNAs via solid-phase synthesis (Fig. 8a). Evaluation of the variants in HeLa cells showed that all three produced a robust translational luminescence signal (Fig. 8b).

Hydrophobic modifications, such as the addition of a 2′-OMe group, progressively decrease the effectiveness of IP-RP HPLC for longer sequences by altering retention patterns, which causes peak broadening and diminished resolution [51]. Consequently, we evaluated the more hydrophobic, photocleavable 5′-C19-nitrobenzyl tag developed by Abe and coworkers (Supplementary Scheme S3) [52]. The tag enhanced RP-HPLC column retention (ΔRt *ca*. 10.2 min) and was quickly and quantitatively cleaved by mild photolysis to yield 5′-monophosphorylated RNA (Supplementary Scheme S4; Supplementary Fig. S10a). This improvement became even more pronounced with the heavily 2′-OMe poly(A) tail modified sequence *M215-025*, where separation proved nearly impossible using the CPRII-tag (Supplementary Fig. S10b).

Finally, we generated a series of four Cap_0 (*m^7^GpppN*) mRNAs with increasing 2′-OMe modifications progressively (5, 10, 20, and 25 units) within their poly(A) tail, a crucial regulatory element that safeguards mRNA from 3′-exonuclease degradation, promotes nuclear export, and enhances translation efficiency by interacting with poly(A)-binding proteins (PABPs) [47, 53]. We observed a direct correlation between the degree of 2′-OMe modification and protein output. The fully modified poly(A) tail (*M215-025*) boosted translation efficiency by 8-fold compared to unmodified mRNA (*M215-native*) 24 h posttransfection (Fig. 8c). Improved mRNA stability, reduced deadenylation rates, and/or preserved PABP binding capacity may account for this observation.

### mRNA characterization by enzymatic digestion and LC-MS analysis

**Figure 9.**
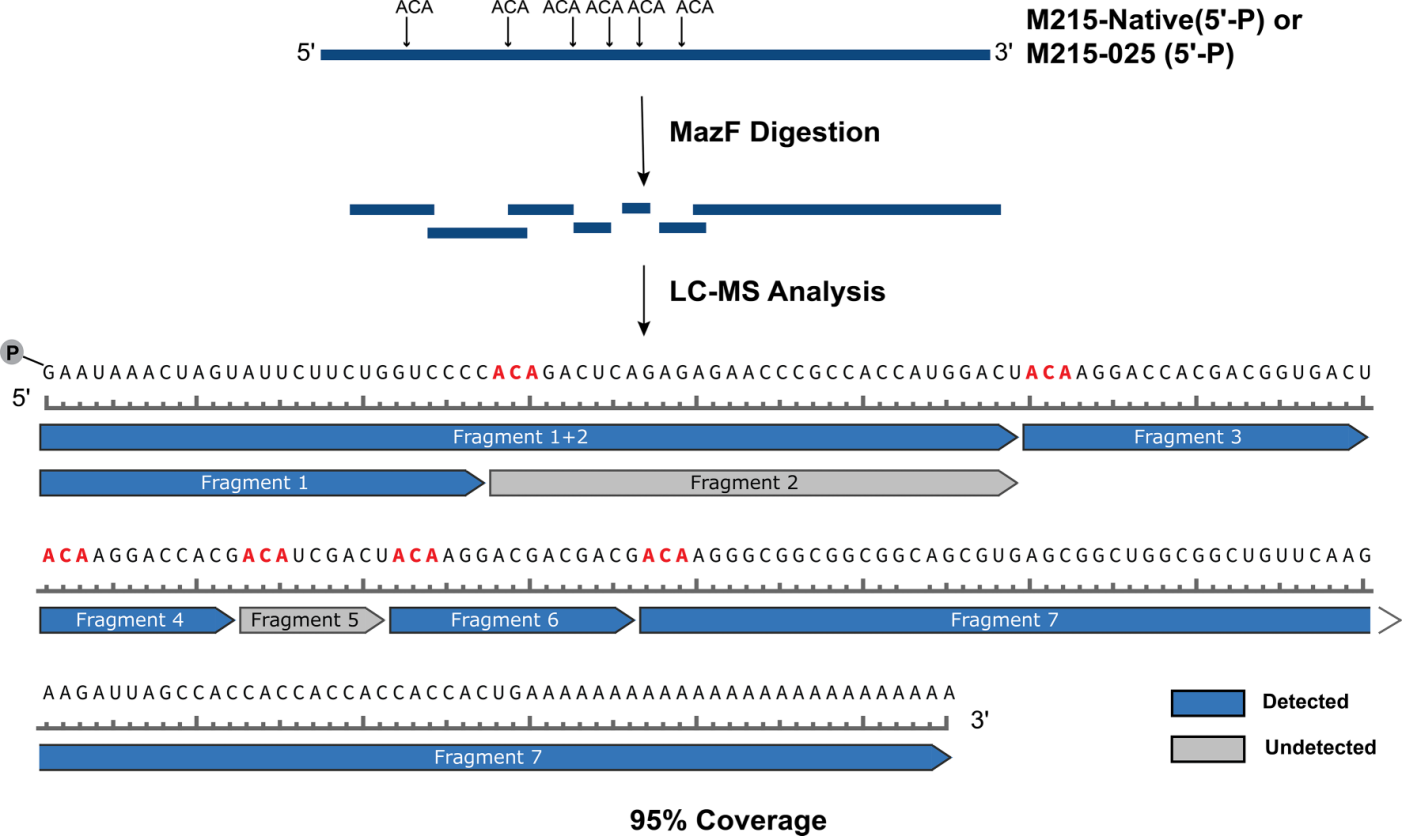
Mapping MazF cleavage fragments from 5′-phosphorylated *M215-native* or *M215-025* mRNA followed by high-resolution ESI-LC–MS analysis. Schematic showed seven detected fragments covering 95% of the RNA sequence following MazF digestion at canonical 5′-ACA-3′ motifs.

We found LC-MS characterization of the full-length long RNAs (>150-nt) challenging, likely a reflection of their large size, increased cation adduction, and complex charge state distribution nature. As a result, we subjected *M215-native* and *M215-025* to nuclease digestion using MazF, a sequence-specific endoribonuclease that cleaves single-stranded RNAs mainly at 5′-ACA sites [54–56], followed by high-resolution LC-MS analysis of the digested shorter fragments (Fig. 9). Analysis of both sets of fragments originating from *M215-native* and *M215-025* resulted in 95% sequence coverage, with successful identification of all expected fragments (Supplementary Table S5) except fragment 5, a 9-nt sequence spanning bases 93–101, likely lost during post-digestion sample cleanup due to its short size. The deconvoluted mass spectra for individual fragments are shown in Supplementary Fig. S11-12. Beyond the expected 5′-ACA cleavage sites, we also identified additional fragments corresponding to cleavage at 5′-AAC sequences, confirming the relaxed substrate specificity of *E. coli* MazF compared to more stringent MazF variants found in other bacterial species (Supplementary Fig. S13).

## Discussion

This study demonstrates that ALE-based solid-phase synthesis is a viable method for producing complex, functionally active RNAs over 200 nts in length. Optimization of RNA synthesis with ALE monomers using *AK26* revealed optimal conditions (4-min coupling, 75 mM monomer), achieving > 99% stepwise coupling efficiency and outperforming TBDMS monomers. This improved efficiency is critical for synthesizing long RNAs: for a 200-nt sequence, boosting the coupling efficiency from 98% and 99.4% increases the theoretical full-length yield from 1.8% to 30.2%. This 17-fold improvement is what makes the process practical instead of merely feasible.

ALE-synthesized RNAs can be deprotected under mild, room-temperature conditions using only a short exposure to amines. After deprotection, RNAs are easily recovered from the solid support by simple aqueous washing, making this approach ideal for large-scale and high-throughput synthesis. For example, high-throughput synthesizers can generate RNAs in a 96-well plate format, and the gentle ALE deprotection chemistry enables fully automated workflows: amine washes remove protecting groups, while final water washes release the crude RNAs from the CPG directly into the 96-well plate, ready for further downstream processing.

We applied these optimized conditions to the successful syntheses of sgRNAs (99 nts), sgRNA tagged with fluorogenic Mango II and Broccoli aptamers (130–170 nts), as well as 5′-capped minimal mRNAs (200–215 nts) that exhibited robust translational activity in both cell-free and cellular systems. Achieving a consistently high coupling efficiency exceeding 99% in the synthesis of 215-nt mRNAs represents a significant milestone, bringing RNA chemical synthesis close to the efficiency of DNA synthesis and approaching the theoretical limits of solid-phase methodology. This achievement addresses the growing demand for synthetic RNA in the 100–300 nt range, including sgRNAs, pegRNAs, RNA aptamers, and mRNA neoantigen vaccine candidates [57].

Over 170 naturally occurring RNA modifications have been identified [58], but only a few are compatible with efficientIVT and amenable to site-specific introduction. ALE-based solid-phase synthesis can accommodate chemical modifications frequently seen in approved oligonucleotide therapeutics [59], making it a viable option for producing modified RNA molecules. As a demonstration, we showed here that the controlled installation of 2′-OMe modifications within the mRNA poly(A) tail produces an 8-fold increase in protein expression, directly illustrating how rationally tuned chemical modifications can improve RNA function within cells. This demonstrates the platform’s potential for site-specific chemical modifications that are difficult or impossible to achieve with enzymatic methods.

Since ALE chemistry is also fully compatible with established RNA ligation strategies, it allows for the rapid assembly of even longer therapeutic mRNAs while systematically exploring modification patterns that could further enhance RNA stability against nucleases, reduce immunogenicity during delivery, and boost translational efficiency. Together, these multifaceted strengths position ALE chemistry as a powerful platform for engineering optimized therapeutic RNAs otherwise inaccessible by purely enzymatic approaches, opening the door to next-generation RNA medicines.

## Supplementary Material

gkaf1525_Supplemental_File

## Data Availability

The data underlying this article are available in the article and in its online supplementary material.
