# Correction to ‘Synthesis of long and functionally active RNAs facilitated by acetal levulinic ester chemistry’

**DOI:** 10.1093/nar/gkag135

**Published:** 2026-02-12

This is a correction to: Zidi Lyu, Adam Katolik, Iqra Yaseen, Adrian A Pater, Francis Robert, Sidong Huang, Keith T Gagnon, Peter J Unrau, Masad J Damha, Synthesis of long and functionally active RNAs facilitated by acetal levulinic ester chemistry, *Nucleic Acids Research*, Volume 54, Issue 2, 27 January 2026, https://doi.org/10.1093/nar/gkaf1525

The name of the fourth co-author has been emended to read: “Adrian A Pater”.

