# Supplementary material for: Synthesis of long and functionally active RNAs facilitated by acetal levulinic ester chemistry
Source: Nucleic Acids Res. 2026 Jan 19;54(2):gkaf1525. doi: 10.1093/nar/gkaf1525 (PMC12812408; doi:10.1093/nar/gkaf1525)
Supplement: gkaf1525_Supplemental_File [file gkaf1525_supplemental_file.docx]

**Supplementary Information**

**Synthesis of long and functionally active RNAs facilitated by acetal levulinic ester (ALE) chemistry**

Zidi Lyu^1^, Adam Katolik^1^, Iqra Yaseen^2^, Adrain A. Pater^3^, Francis Robert^4^, Sidong Huang^4^, Keith T. Gagnon^3^, Peter J. Unrau^2^, and Masad J. Damha^1,^*

^1^ Department of Chemistry, McGill University, Montreal, Quebec, H3A 0B8, Canada

^2^ Department of Molecular Biology and Biochemistry, Simon Fraser University, Burnaby, British Columbia, V5A 1S6, Canada

^3^ Department of Biochemistry, Wake Forest University School of Medicine, Winston-Salem, North Carolina, NC 27157, United States

^4^ Department of Biochemistry, Rosaline & Morris Goodman Cancer Institute, McGill University, Montreal, Quebec, H3G 1Y6, Canada

* To whom correspondence should be addressed. Email: [masad.damha@mcgill.ca](mailto:masad.damha@mcgill.ca)

Table of Contents

[**1.** **General Materials and Instruments** 3](#_Toc216630316)

[**2.** **General Experimental Procedures** 4](#_Toc216630317)

[**2.1. Deprotection of oligonucleotide made by TBDMS monomers** 4](#_Toc216630318)

[**2.2. Denaturing polyacrylamide gel purification and analysis** 4](#_Toc216630319)

[**2.3. Chemical synthesis of 5'-monophosphorylated RNA with CPR II** 5](#_Toc216630320)

[**Scheme S1.** Scheme of 5'-monophosphorylated mRNA using Chemical Phosphorylation Reagent II (CPRII). 5](#_Toc216630321)

[**2.4 Chemical capping of 5'-monophosphorylated mRNA** 5](#_Toc216630322)

[**Scheme S2**. Chemical capping of mRNA using Im-m^7^GDP in DMSO (1). 5](#_Toc216630323)

[**2.5 Chemical synthesis of 5'-monophosphorylated RNA with C19-nitrobenzyl tag** 6](#_Toc216630324)

[**Scheme S3.** Synthesis scheme of C19-nitrobenzyl phosphoramidite **4**. 7](#_Toc216630325)

[**Scheme S4**. Scheme of 5'-monophosphorylation using C19-nitrobenzyl phosphoramidite 7](#_Toc216630326)

[**3.** **Supplementary Tables** 7](#_Toc216630327)

[**Table S1**. RNA Sequences made via 2'-*O*-ALE phosphoramidite in this study 8](#_Toc216630328)

[**Table S2**. Sequence of 52-base pair double-stranded DNA target for sgRNA cleavage assays 9](#_Toc216630329)

[**Table S3**. LC-MS characterization and yield comparison of fluorescently tagged RNA complexes synthesized using ALE versus TBDMS monomers 9](#_Toc216630330)

[**Table S4**. Effect of solid support pore size on synthesis yields of fluorescently tagged sgRNAs more than 150-nt 9](#_Toc216630331)

[**Table S5**. High-resolution ESI-LC-MS analysis of fragments from MazF digestion of *M215-native* and *M215-025* mRNAs 10](#_Toc216630332)

[**4.** **Supplementary Figures** 10](#_Toc216630333)

[**Figure S1**. Systematic optimization of ALE solid-phase synthesis conditions using 26-nt RNA (*AK26*) 10](#_Toc216630334)

[**Figure S2**. High resolution ESI-LC-MS evaluation of 11](#_Toc216630335)

[**Figure S3**. Incorporation of Mango II and/or Broccoli aptamers into different regions at the sgRNA 12](#_Toc216630336)

[**Figure S4**. Analysis of crude and purified fluorogenic sgRNAs. 13](#_Toc216630337)

[**Figure S5-S7**. High resolution ESI-LC-MS characterization of Mango II-tagged sgRNAs. 14](#_Toc216630338)

[**Figure S8**. Characterization of the 200-nt minimal mRNA construct (*M200*). 17](#_Toc216630339)

[**Figure S9**. 5'-Capping efficiency evaluated with *AK26* sequence 18](#_Toc216630340)

[**Figure S10**. Comparative reverse phase HPLC (RP-HPLC) purification of 5′-phosphorylated mRNA using CPRII or C19-nitrobenzyl tags 19](#_Toc216630341)

[**Figure S11**. High-resolution LC-MS analysis of MazF cleavage products from *M215-native* mRNA 20](#_Toc216630342)

[**Figure S12**. High-resolution LC-MS analysis of MazF cleavage products from *M215-025* mRNA 21](#_Toc216630343)

[**Figure S13**. Alternative MazF cleavage products from *M215-native* RNA 22](#_Toc216630344)

[**5.** **References** 23](#_Toc216630345)

# **General Materials and Instruments**

All starting materials and chemicals were sources from Sigma-Aldrich or Thermo Fisher Scientific without further purification. HPLC grade solvents were purchased from Thermo Fisher Scientific. All phosphoramidites, controlled pore glass (CPG) solid supports, and reagents for oligonucleotide solid-phase synthesis were purchased from ChemGenes Corporation and Glen Research with details provided in the Materials and Methods section. MerMade 12, K&A S-4-LC, or K&A S-8 DNA/RNA synthesizer (Sierra BioSystems) were used for oligonucleotide synthesis. For the K&A synthesizer, all amidites/reagents were pressurized under 0.04 MPa Argon (Linde Gas), the valves were kept closed with the aid of air compressor of 0.4 MPa (Panther Silent Air Compressor - P30TC). Agilent 1290 Infinity II preparative system was used with the following configuration: prep binary pump G7161B, open-bed sampler and collector G7158B, diode array detector G7115A, and multicolumn thermostat G7116A. The SpeedVac vacuum concentrator system kit was obtained from Thermo Fisher Scientific with model SRF110P1-115.

# **General Experimental Procedures**

## **2.1. Deprotection of oligonucleotide made by TBDMS monomers**

For RNA made via TBDMS phosphoramidites in 1 µmol scale, the cleavage from CPG and base deprotection was carried out in a 1 mL solution of 3:1 ammonium hydroxide (28.0% to 30.0% w/w, Fisher Scientific) in ethanol. The tube was put on a thermal shaker maintained at 55°C for 16 hours, after which the solution was evaporated using a SpeedVac vacuum concentrator. DNase/RNase Free water (3 x 400 μL) was added, and the supernatant was filtered through 0.2 μm sterile filter to yield the crude 2'-TBDMS protected RNA. The RNA was then spin dried in the SpeedVac. To remove the remaining TBDMS groups on the RNA, 300 µL of a “cocktail” solution of N-methylpyrrolidone (NMP, Thermo Fisher Scientific) / Triethylamine (TEA, Sigma) / Triethylamine trihydrofluoride (TREAT-HF, Thermo Fisher Scientific) was added. The solution was prepared by adding NMP, TEA, and TREAT-HF in 3:4:6 ratio in order. The mixture was shaken at 65°C for 90 min. The reaction was quenched by adding 50 μL of 3 M sodium acetate (pH 5.5). The RNA was recovered by adding 1 mL of cold butanol (-20°C from the freezer) to the precipitate the RNA. The sample was left on dry ice for 30 min, then centrifuged for 5 min at 12,000 × g to recover the RNA as pellets at the bottom of the tube. The supernatant was carefully decanted or pipetted out and leaving the pellets behind. Add another 1 mL cold butanol to repeat the process. The RNA pellet was dried in SpeedVac for at least 20 min. The recovered RNA can then be re-dissolved in DNase/RNase Free water for further downstream purification or analytical procedures.

## **2.2. Denaturing polyacrylamide gel purification and analysis**

Oligonucleotides were purified by denaturing polyacrylamide gel electrophoresis (PAGE) in plate size of 18 × 16 cm. A solution of denaturing polyacrylamide gel was prepared by mixing appropriate volume of 40% (w/v) acrylamide/bis-acrylamide solution (19:1, Bishop Canada) with urea to final concentration of 7M, 1 × Tris-Borate-EDTA (TBE) buffer, and DEPC-treated MilliQ Water to desired final volume. Polymerization was initiated by adding 0.1% v/v tetramethylethylenediamine (TEMED, Sigma) and specific amount of 10% (w/v) ammonium persulfate (Sigma). Only 1/3 or 1/4 of 1 μmol scale synthesis was purified on a single 18 × 16 cm gel with a 1.5-cm thick comb. Samples were prepared in denaturing gel loading buffer containing 45% formamide (Bishop Canada) and 10 mM EDTA (Sigma), followed by heating at 95°C for 5 min to denature. The gel apparatus was cooled by running water (Hoefer SE600 Series Gel Apparatus). The samples were then loaded into the pre-run wells of the gel. Electrophoresis was performed in 1 × TBE buffer at constant current until adequate separation was achieved. Next the gel was examined under UV shadowing, and the major (slowest moving) band was excised into a sterile 10 mL sample tube. The gel was crushed into smaller pieces, then soaked in 0.3 M NaCl solution. Next, the tube, which contained the gel, was frozen with liquid nitrogen and placed at 4 °C overnight. The next day, the supernatant was carefully pipetted out and filtered through a sterile 0.45 μm membrane. The filtrate was split into 400-μL portions into a 1.5 mL microcentrifuge tube and 1 mL of ethanol was added to each. The RNA was recovered by incubation at -20°C for at least 2 hours, followed by centrifugation at 4°C at 12,000 × g for 15 min to collect the RNA pellet. The pellet was re-dissolved in DNase/RNase free water and further desalted with a Glen Gel-PAK^TM^ 2.5 Desalting column (Glen Research) according to the manufacture’s protocol. For oligonucleotides characterized by denaturing PAGE, the electrophoresis was carried out in Mini-PROTEAN Tetra Vertical Electrophoresis Cell (Bio-Rad) with the same receipt of polyacrylamide solution described above. The gel was stained in 25 mL of 2× SYBR^TM^ Gold Nucleic Acid Gel Stain (Invitrogen, dilution with water from a 10,000× Concentrate in DMSO) for 20 min at room temperature. The RNA was visualized with a gel imaging system (Gel Doc^TM^ XR+ system with Image Lab^TM^ Software, Bio-Rad).

## **2.3. Chemical synthesis of 5'-monophosphorylated RNA with CPR II**

### **
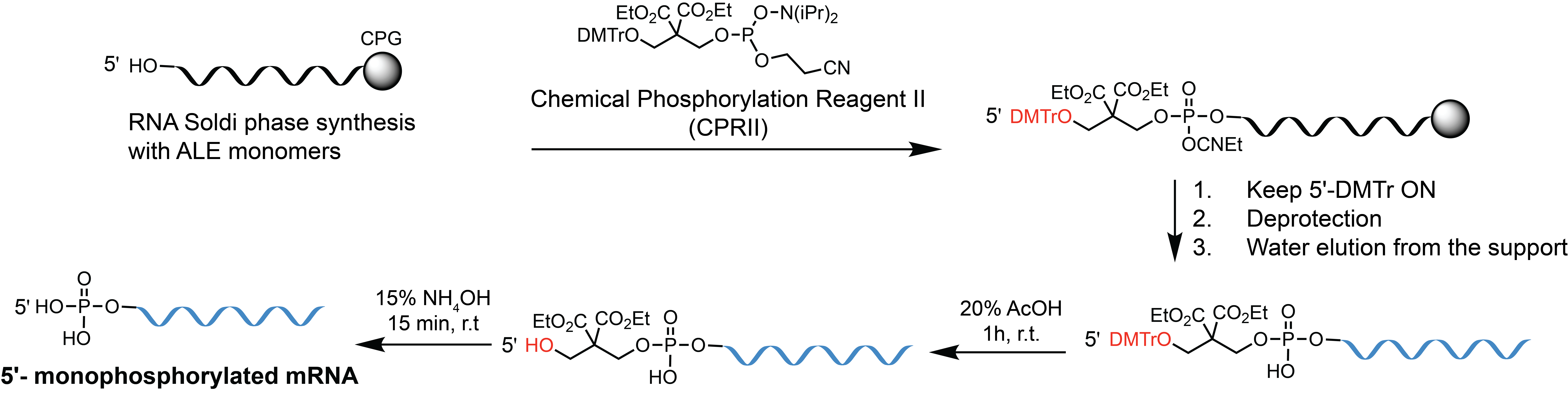
Scheme S1.** Scheme of 5'-monophosphorylated mRNA using Chemical Phosphorylation Reagent II (CPRII).

Chemical Phosphorylation Reagent II (CPRII, Glen Research 10-1901) was coupled to the 5'-terminus during solid-phase synthesis with retention of the final DMTr group as a hydrophobic handle for subsequent reverse-phase HPLC purification. The DMTr group remained stable under the deprotection conditions used for ALE monomers. Following deprotection, RNA was eluted from the solid support with water and subjected to reverse-phase HPLC purification to collect fractions containing full-length product. RNA was precipitated from HPLC fractions by adding 0.1 volume of 3 M sodium acetate buffer (pH 5.5) followed by 1 volume of cold isopropanol. The mixture was incubated at -20°C for at least 1 h, then centrifuged at 12,000 × g at 4°C to recover the RNA pellet. After vacuum drying the pellet, the purified RNA was treated with 20% acetic acid for 1 h at room temperature to remove the DMTr group, followed by treatment with 10% ammonium hydroxide for 15 min at room temperature to cleave the CPRII side chain and generate the 5'-monophosphorylated mRNA.

## **
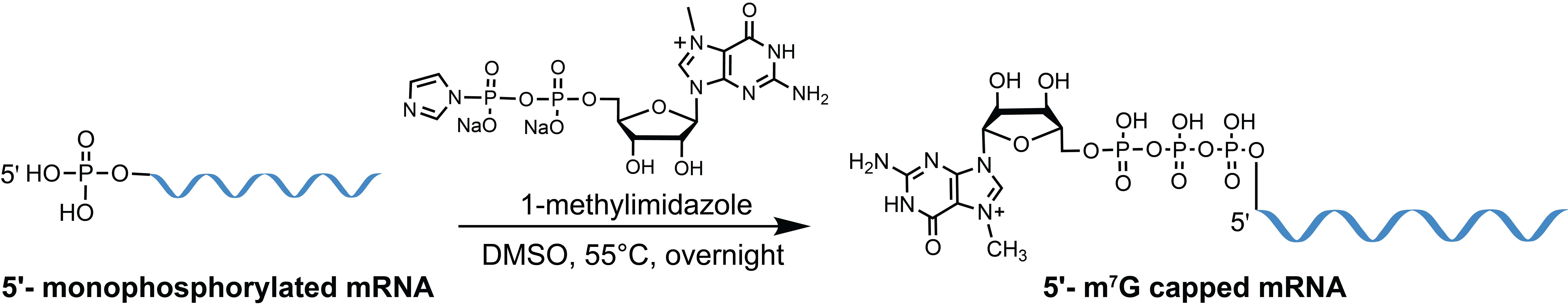
2.4 Chemical capping of 5'-monophosphorylated mRNA**

### **Scheme S2**. Chemical capping of mRNA using Im-m^7^GDP in DMSO (1).

The 5'-monophosphorylated mRNA was subjected to chemical capping reaction to yield 5'-capped mRNA as described before (1). 5'-monophosphorylated RNA (2 nmol) was lyophilized with 20 μL of 100 mM aqueous CaCl_2_, and redissolved in 84 μL of anhydrous dimethyl sulfoxide (DMSO). 100 µL of 20 mM Im-m⁷GDP sodium salt (Hongene R2-066) in DMSO was added, followed by 16 μL of 1-methylimidazole (Sigma). The mixture was shaken at 55°C overnight. The reaction was quenched by adding 200 μL of 3 M sodium acetate (pH 5.5) and the RNA was recovered by precipitation with cold butanol. The RNA was re-dissolved in 500 μL of DNase/RNase-free water and desalted using a Glen Gel-PAK™ 2.5 Desalting column (Glen Research) according to the manufacturer's protocol to remove impurities from the reaction.

## **
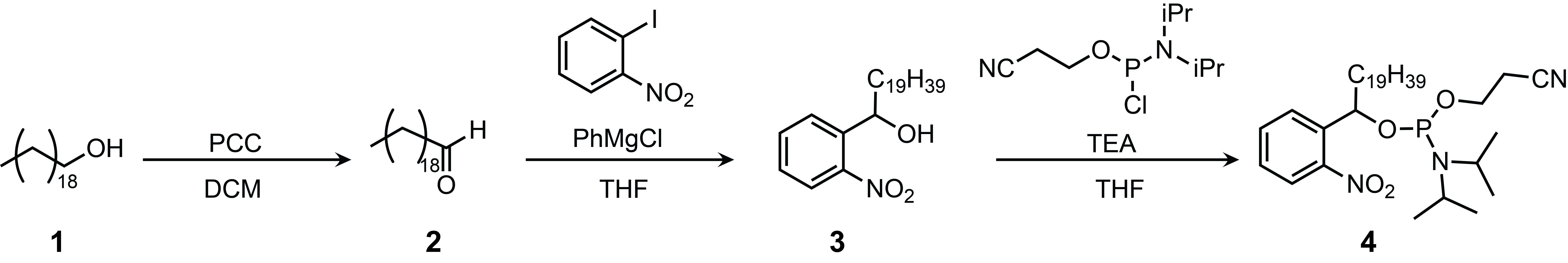
2.5 Chemical synthesis of 5'-monophosphorylated RNA with C19-nitrobenzyl tag**


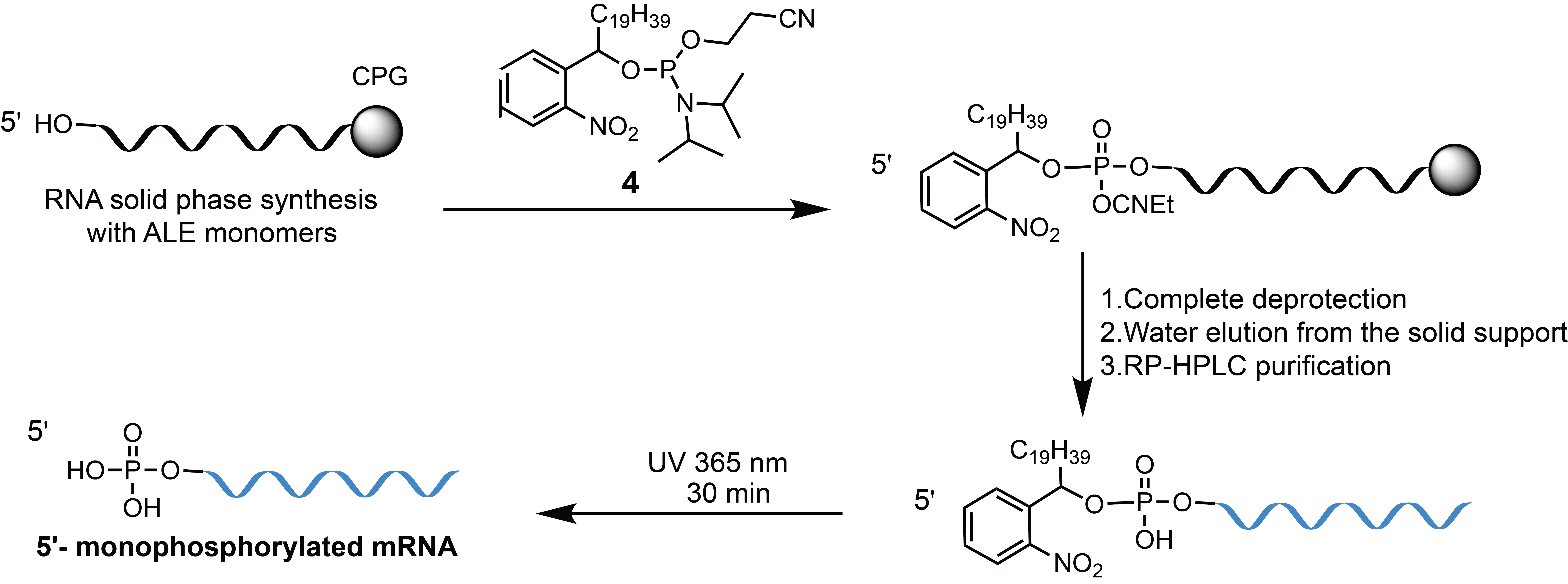
**Scheme S3.** Synthesis scheme of C19-nitrobenzyl phosphoramidite **4**. The compounds were synthesized according to previously published procedures (2).

**Scheme S4**. Scheme of 5'-monophosphorylation using C19-nitrobenzyl phosphoramidite **4**.

The C19-nitrobenzyl phosphoramidite **4** (0.15 M in 1:9 v/v DCM/ACN, 15 min coupling time) was coupled to the 5'-terminus during solid-phase synthesis. The photocleavable tag remained stable under the deprotection conditions used for ALE monomers. Following deprotection, RNA was eluted from the solid support with water and subjected to reverse-phase HPLC purification to collect fractions containing the full-length product. RNA was precipitated from the HPLC fractions by adding 0.1 volume of 3 M sodium acetate buffer (pH 5.5) followed by 1 volume of cold isopropanol. The mixture was incubated at -20°C for at least 1 h, then centrifuged at 12,000 × g at 4°C to recover the RNA pellet. Purified RNA was redissolved in water and transferred to transparent 24-well plates (100 μL per well), the C19-nitrobenzyl tag was deprotected by UV irradiation at 365 nm for 30 min. Following the photo-deprotection, the 5'-monophosphorylated RNA was pooled into a 1.5-mL microcentrifuge tube and desalted with Glen Gel-PAK™ 2.5 Desalting column (Glen Research) according to the manufacturer's protocol.

# **Supplementary Tables**

| RNA Sequence | Length(nt) | Sequence (5 ' - 3 ') |
| --- | --- | --- |
| AK26 | 26 | AAGUUAAAAUAAGGCUAGUCCGUUAU^2'OMe^ |
| AK99 | 99 | GGGCGAGGAGCUGUUCACCGGUUUUAGAGCUAGAAAUAGCAAGUUAAAAUAAGGCUAGUCCGUUAUCAAACUUGAAAA AGUGGCACCGAGUCGGUGCUU^2'OMe^ |
| GHL1-5 | 109 | GGAUAACUCAAUUUGUAAAAAAGUUUUAGAGCUACGAAGGAGAGGAGAGGAAGAGGAGAGUAGCAAGUUAAAAUAAGGCUAGUCCGUUAUCAACUUGAAAAAGUGUUGG |
| GHL3-10 | 119 | GGAUAACUCAAUUUGUAAAAAAGUUUUAGAGCUAGAAAUAGCAAGUUAAAAUAAGGCUAGUCCGUUAUCACACUUACGUAGAAGGAGAGGAGAGGAAGAGGAGAUACGUAAGUGGUUGG |
| G3P-9 | 133 | GGAUAACUCAAUUUGUAAAAAAGUUUUAGAGCUAGAAAUAGCAAGUUAAAAUAAGGCUAGUCCGUUAUCAACUUGAAAAAGUGUUGGUCCCACUUACGUAGAAGGAGAGGAGAGGAAGAGGAGAUACGUAAGU |
| MB160 | 160 | GGAUAACUCAAUUUGUAAAAAAGUUUUAGAGCUAGAGACGGUCGGGUCCAGAUAUUCGUAUUCGUAUCUGUCGAGUAGAGUGUGGGCUCUAGCAAGUUAAAAUAAGGCUAGUCCGUUAUCAACUUCGAAGGAGAGGAGAGGAAGAGGAGAGAAGUGUUGG |
| MB170 | 170 | GGAUAACUCAAUUUGUAAAAAAGUUUUAGAGCUAGAGACGGUCGGGUCCAGAUAUUCGUAUUCGUAUCUGUCGAGUAGAGUGUGGGCUCUAGCAAGUUAAAAUAAGGCUAGUCCGUUAUCACACUUACGUAGAAGGAGAGGAGAGGAAGAGGAGAUACGUAAGUGGUUGG |
| M200 | 200* | GAAUAAACUAGUAUUCUUCUGGUCCCCACAGACUCAGAGAGAACCCGCCACC**AUG**GACUACAAGGACCACGACGGUGACUACAAGGACCACGACAUCGACUACAAGGACGACGACGACAAGUGAGGCGGCGGCGGCAGCGUGAGCGGCUGGCGGCUGUUCAAGAAGAUUAGC**UGA**AAAAAAAAAAAAAAAAAAAAAAAAA |
| M215 | 215* | GAAUAAACUAGUAUUCUUCUGGUCCCCACAGACUCAGAGAGAACCCGCCACC**AUG**GACUACAAGGACCACGACGGUGACUACAAGGACCACGACAUCGACUACAAGGACGACGACGACAAGGGCGGCGGCGGCAGCGUGAGCGGCUGGCGGCUGUUCAAGAAGAUUAGCCACCACCACCACCACCAC**UGA**AAAAAAAAAAAAAAAAAAAAAAAAA |

**Table S1**. RNA Sequences made via 2'-*O*-ALE phosphoramidite in this study. *The length of *M200* and *M215* excludes the count of 5'-cap structure (m⁷Gppp).

| **dsDNA Target for Cleavage Assay (5'-3')** | |
| --- | --- |
| Complementary Strand | TAATGAATTCCCCAATACCCTTT**\|**TTTACAAATTGAGTTATCCGTTCATATAA |
| Non-Complementary Strand | TTATATGAACGGATAACTCAATTTGTAAA**\|**AAAGGGTATTGGGGAATTCATTA |

**Table S2**. Sequence of 52-base pair double-stranded DNA target for sgRNA cleavage assays. The target sequence is designed for cleavage assays using Mango II tagged sgRNAs. Underlined nucleotides indicate the gRNA-complementary region on the target strand, with the corresponding region on the non-target strand. Red nucleotides represent the PAM sequence recognized by Cas9, and the red bold line denotes the Cas9 cleavage site.

| **Sequence** | **Theoretical MW(Da)** | **Observed MW(Da)** | **Yield-ALE (nmol)** | **Yield-TBDMS (nmol)** |
| --- | --- | --- | --- | --- |
| GHL1-5 | 35501.2083 | 35501.0961 | 21.3 | 12.5 |
| GHL3-10 | 38708.0963 | 38708.8125 | 31.8 | 6.2 |
| G3P-9 | 43168.7443 | 43168.0000 | 7.3 | 5.3 |

**Table S3**. LC-MS characterization and yield comparison of fluorescently tagged RNA complexes synthesized using ALE versus TBDMS monomers. The observed molecular weights obtained from high resolution LC-MS analysis demonstrated the product integrity for all constructs. ALE chemistry consistently achieved higher yields after purification compared to TBDMS synthesis across all sequences tested.

| **Sequence** | **Yield-2000** **Å (nmol)** | **Yield-3000** **Å (nmol)** |
| --- | --- | --- |
| MB-160 | 5.4 | 8.0 |
| MB-170 | 3.8 | 16.0 |

**Table S4**. Effect of solid support pore size on synthesis yields of fluorescently tagged sgRNAs more than 150-nt. Isolated yields of Broccoli and Mango II aptamer-tagged sgRNAs (*MB-160*, 160-nt; *MB-170*, 170-nt) synthesized using ALE monomers on succinyl-linked solid supports with 2000 Å and 3000 Å pore sizes. Larger pore size (3000 Å) provided improved yields for both constructs.

| **Fragment** | **Position**  **(5' - 3')** | **Length (nt)** | **Theoretical mass (Da)** | **Observed mass**  **(Da)-*M215*** | | **Observed Mass**  **(Da)-*M215-025*** | |
| --- | --- | --- | --- | --- | --- | --- | --- |
| 1 | 1-27 | 27 | 8653.0404 | | 8653.8705 | | 8653.8125 |
| 1+2 | 1-59 | 59 | 18970.3127 | | 18970.8125 | | 18970.0000 |
| 3 | 60-80 | 21 | 6815.9506 | | 6815.8414 | | 6815.7813 |
| 4 | 81-92 | 12 | 3900.5701 | | 3900.5169 | | 3900.4938 |
| 5 | 102-116 | 15 | 4919.7174 | | 4919.6500 | | 4919.6438 |
| 6 | 117-215 | 99 | 32152.7197 (*M215*)  32503.3353 (*M215-025*) | | 32152.4310 | | 32503.0625 |
| S1 | 1-42 | 42 | 13564.0588 | | 13563.8412 | | 13563.5112 |
| S2 | 43-59 | 17 | 5403.7333 | | 5403.6810 | | 5403.6438 |
| S3 | 7-42 | 36 | 11515.8704 | | 11515.0457 | | Not observed |

**Table S5**. High-resolution ESI-LC-MS analysis of fragments from MazF digestion of *M215-native* and *M215-025* mRNAs. Fragments 1–6 originate from canonical 5′-ACA-3′ cleavage sites, while S1–S3 result from non-canonical adenosine cuts at 5′-AAC-3′ motifs. Monoisotopic exact masses are reported for fragments <10 kDa, and molecular weights for fragments >10 kDa.

# **Supplementary Figures**


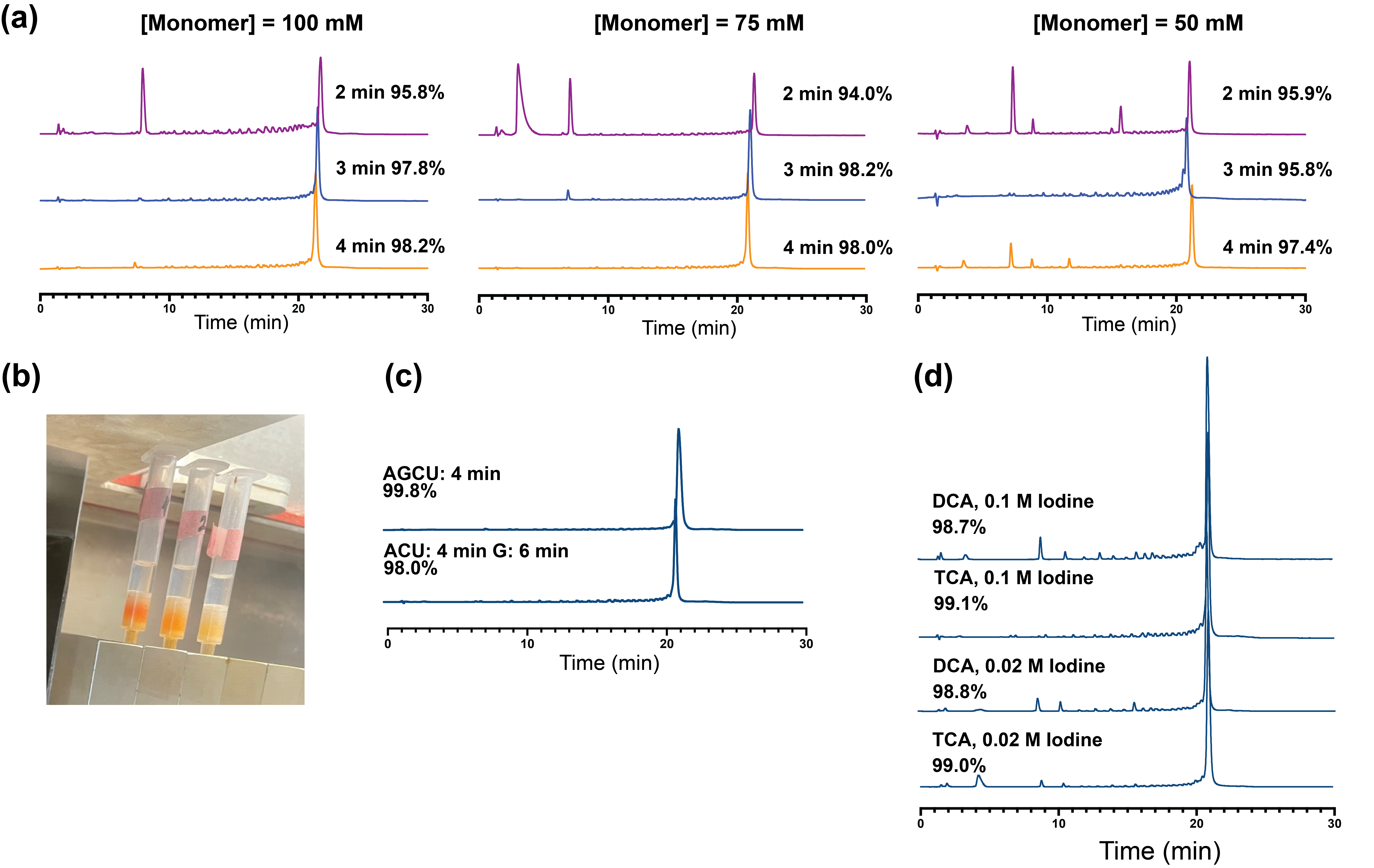


**Figure S1**. Systematic optimization of ALE solid-phase synthesis conditions using 26-nt RNA (*AK26*). **(a)** Ion-exchange HPLC chromatograms showing the effect of monomer concentration (100, 75, and 50 mM) and coupling time (2, 3, and 4 min) on coupling efficiency. Coupling efficiency values were indicated for each condition, with optimal performance achieved at 75 mM concentration and 4 min coupling time. **(b)** Final detritylation color image demonstrating visual assessment of yield during synthesis at 100 mM monomer concentration with 4-, 3-, and 2-minute coupling times (left to right). **(c)** Comparison of HPLC profiles for *AK26* synthesized with standard coupling times versus extended G-coupling protocols, showing no improved efficiency. **(d)** Evaluation of different detritylation and oxidation reagents, demonstrating TCA with 0.1 M iodine as the optimal condition.

**
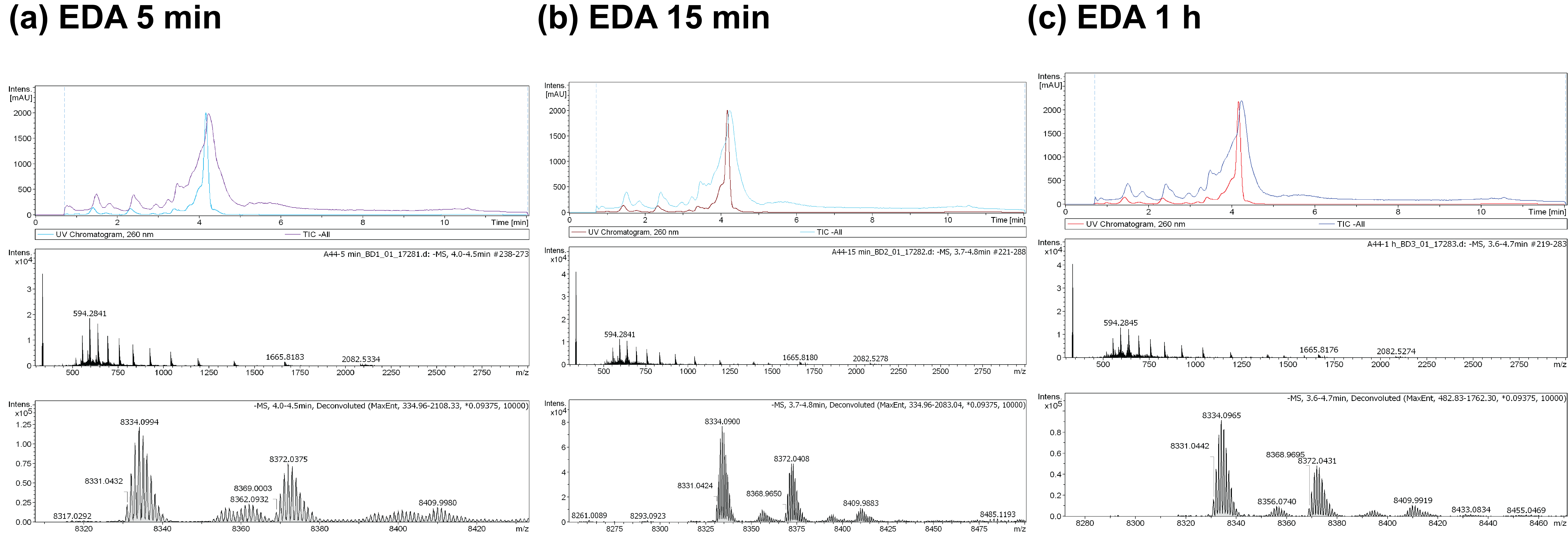
**

**Figure S2**. High resolution ESI-LC-MS evaluation of ethylenediamine (EDA) deprotection completion for *AK26* RNA. Each panel shows the UV chromatogram at 260 nm (top), total ion chromatogram (middle), and deconvoluted mass spectrum (bottom). Theoretical monoisotopic mass of the fully deprotected product is 8331.1472 Da. The deconvoluted mass spectrum showed EDA treatment for **(a)** 5 min: [M] 8331.0432 Da, [M+K]^+^ 8369.0003 Da; **(b)** 15 min: [M] 8331.0424 Da, [M+K]^+^ 8368.9650 Da; **(c)** 1 h: [M] 8331.0442 Da, [M+K]^+^ 8368.9695 Da.

| Sequence | Aptamer insertion |
| --- | --- |
| GHL1-5 | Mango II insertion and 5 bps in Helix Loop 1 |
| GHL3-10 | Mango II insertion and 10 bps in Helix Loop 3 |
| G3P-9 | Mango II insertion and 9 bps in helix on the 3' end, 4 nt extension |
| MB160 | Broccoli (HL1) Mango II (HL3) gRNA, 5 bps in MII stem |
| MB170 | Broccoli (HL1) Mango II (HL3) gRNA, 10 bps in MII stem |


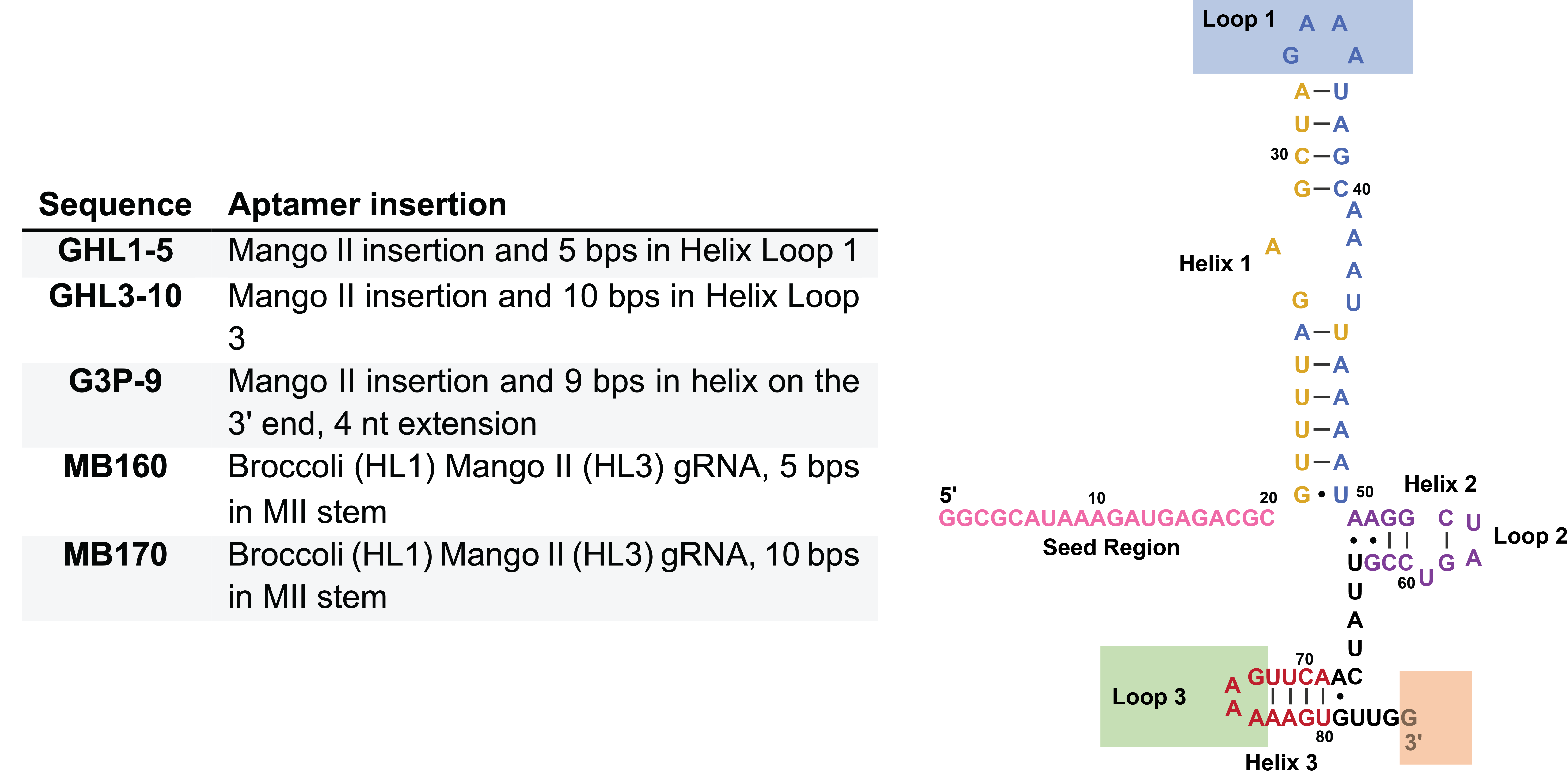


**Figure S3**. Incorporation of Mango II and/or Broccoli aptamers into different regions at the sgRNA, generating different fluorogenic sgRNAs (*GHL1-5*, *GHL3-10*, *G3P-9*, *MB-160*, and *MB-170*).





**Figure S4**. Analysis of crude and purified fluorogenic sgRNAs. **(a)** 10% denaturing PAGE analysis of fluorogenic sgRNAs *GHL1-5*, *GHL3-10*, and *G3P-9* comparing crude and purified products synthesized from ALE chemistry. **(b)** 10% denaturing PAGE analysis of dual-tagged constructs *MB-160* (160-mer) and *MB-170* (170-mer) synthesized on 3000 Å CPG pore size. **(c)** Analytical ion-exchange HPLC chromatograms for dual-tagged sgRNAs *MB-160* and *MB-170* synthesized on 3000 Å CPG. PAGE gel-purified chromatograms showed the removal of failure strands that contributed to the peak broadening in the crude samples, resulting in sharper peaks after gel purification with isolated yields of 8.0 nmol and 16.0 nmol, respectively.

## **Figure S5-S7**. High resolution ESI-LC-MS characterization of Mango II-tagged sgRNAs.

**Figure S5.** High resolution ESI-LC-MS analysis of the Mango II tagged RNAs, *GHL1-5.* Theoretical MW: 35501.2083. Top panel: UV (260 nm) chromatogram overlaid with total ion chromatogram from ion-pairing reverse-phase HPLC. Middle panel: raw MS spectrum showing charge envelope. Bottom panel: deconvoluted mass displaying 35501.0961 Da for the full-length product.

**Figure S6.** High resolution ESI-LC-MS analysis of the Mango II tagged RNAs, *GHL3-10.* Theoretical MW: 38708.0963. Top panel: UV (260 nm) chromatogram overlaid with total ion chromatogram from ion-pairing reverse-phase HPLC. Middle panel: raw MS spectrum showing charge envelope. Bottom panel: deconvoluted mass displaying 38708.8125 Da for the full-length product.

**Figure S7.** High resolution ESI-LC-MS analysis of the Mango II tagged RNAs, *G3P-9.* Theoretical MW: 43168.7443. Top panel: UV (260 nm) chromatogram overlaid with total ion chromatogram from ion-pairing reverse-phase HPLC. Middle panel: raw MS spectrum showing charge envelope. Bottom panel: deconvoluted mass displaying 43168.0000 Da for the full-length product.


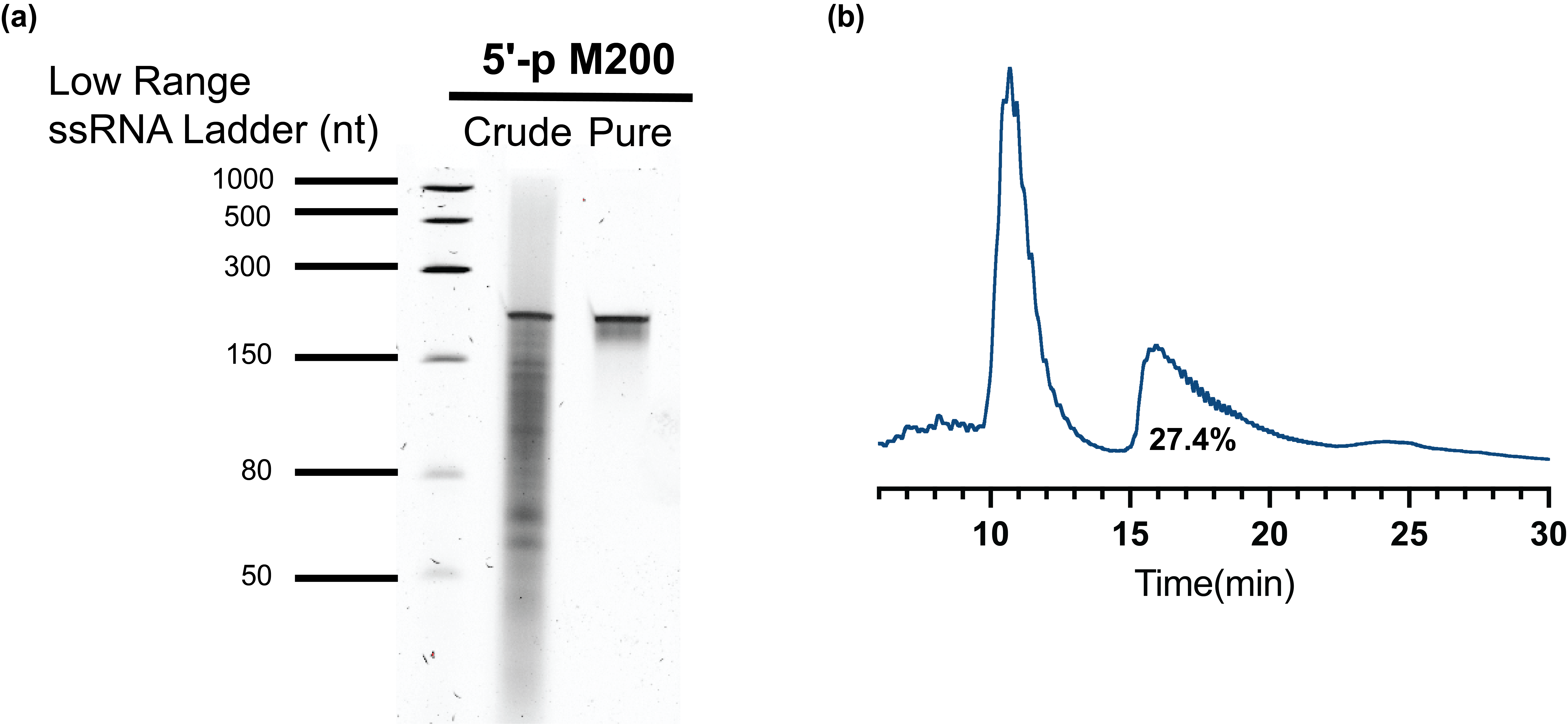


**Figure S8**. Characterization of the 200-nt minimal mRNA construct (*M200*). **(a)** 6% denaturing PAGE analysis comparing crude and purified *M200*: the crude lane shows a smear of truncated species, while the purified lane displays a discrete band around 200-nt by comparing with the ladder. **(b)** Reverse-phase HPLC chromatogram of *M200*. The second major peak corresponds to the CPRII-tagged full-length product, comprising 27.4% of total area.

**
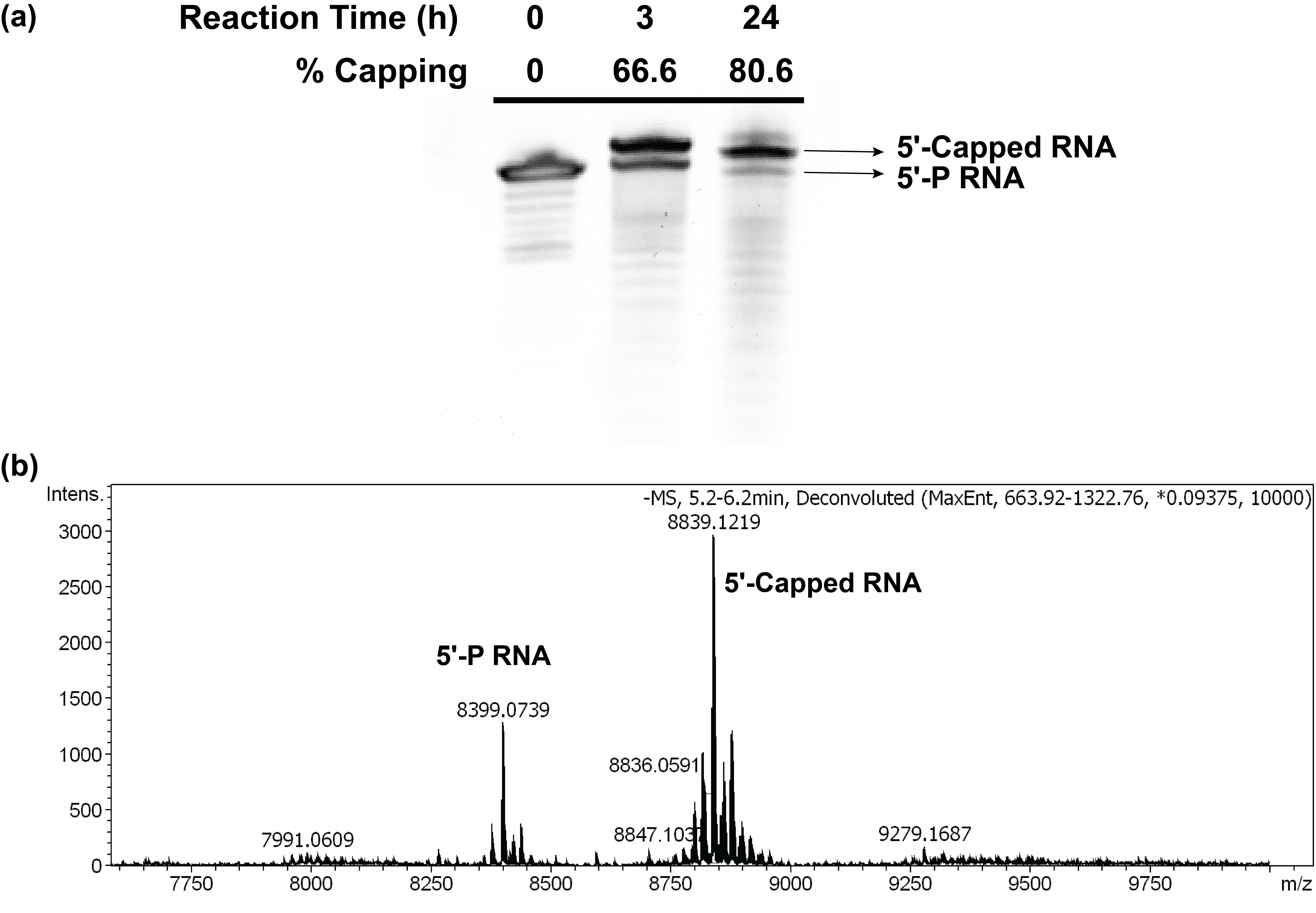
**

**Figure S9**. 5'-Capping efficiency evaluated with *AK26* sequence (5' AAGUUAAAAUAAGGCUAGUCCGUUAU-3'). **(a)** 15% denaturing PAGE analysis showing conversion of 5′-monophosphorylated (5′-P) *AK26* to 5'-capped product over 24 hours, with capping efficiency reaching 66.6% at 3 hours and 80.6% at 24 hours. **(b)** High-resolution LC–MS analysis of the 24-hours capping reaction product displaying deconvoluted mass spectrum with peaks corresponding to unreacted 5′-phosphorylated RNA (8399.0739 Da) and 5′-capped product (8839.1219 Da). Theoretical exact masses: 5′-P *AK26*, 8397.0992 Da; 5′-capped *AK26*, 8837.1359 Da.





**Figure S10**. Comparative reverse phase HPLC (RP-HPLC) purification of 5′-phosphorylated mRNA using CPRII or C19-nitrobenzyl tags. **(a)** ***M215-native*** mRNA: RP-HPLC chromatograms showed DMTr-ON phosphorylation using CPRII (left) or C19-nitrobenzyl tag (right), both showing separation of failed-strands from full-length product, while C19-nitrobenzyl yielded cleaner and less degraded product after post-purification deprotection from the denaturing 6% PAGE gel analysis (inset). **(b) *M215-025*** mRNA**:** C19-nitrobenzyl tagged mRNA achieved baseline resolution of failed strands (RT 5.78 min) and tagged full-length product (RT 13.58 min), whereas CPRII showed poor separation.

**
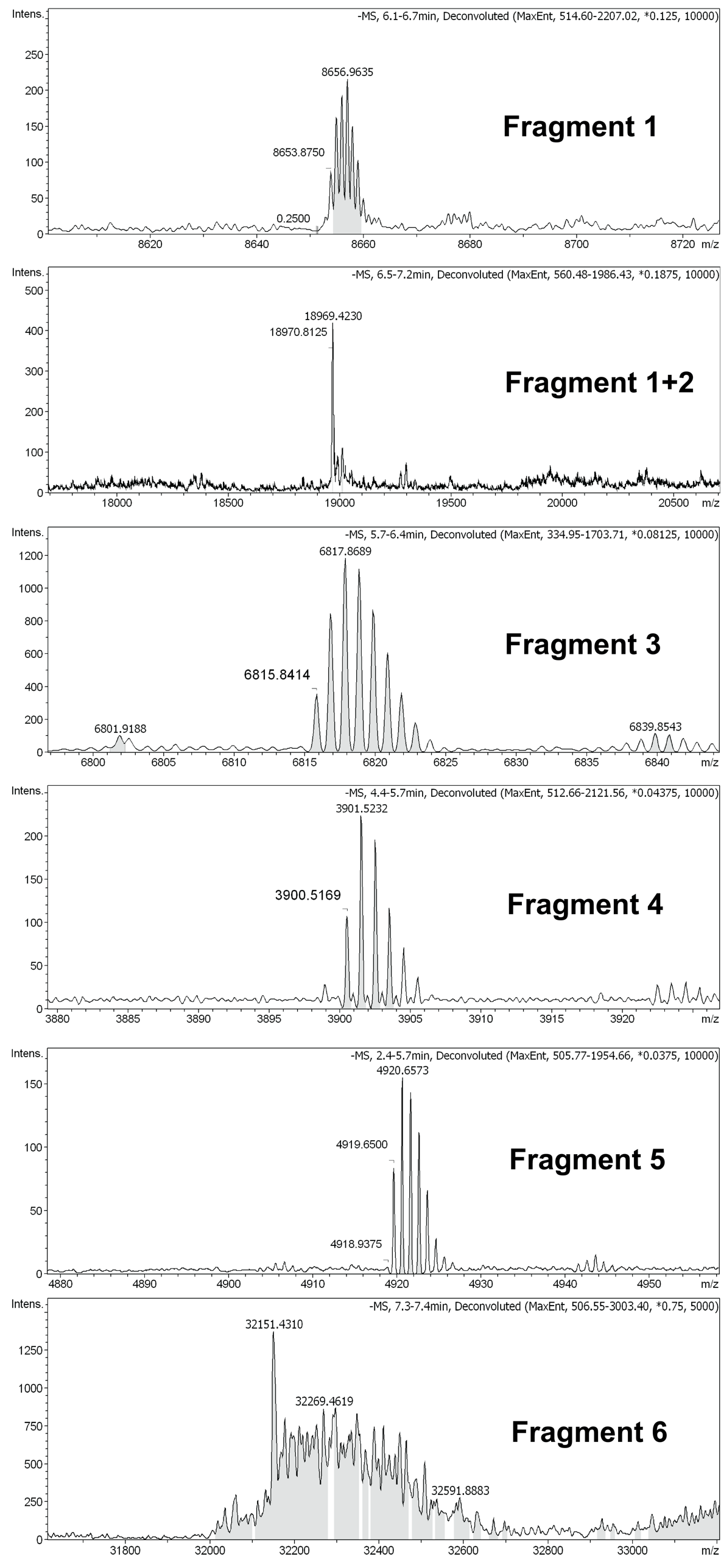
**

**Figure S11**. High-resolution LC-MS analysis of MazF cleavage products from *M215-native* mRNA. Deconvoluted mass spectra of individual RNA fragments generated by *E. coli* MazF endonuclease digestion, with observed masses and fragment assignments.

**
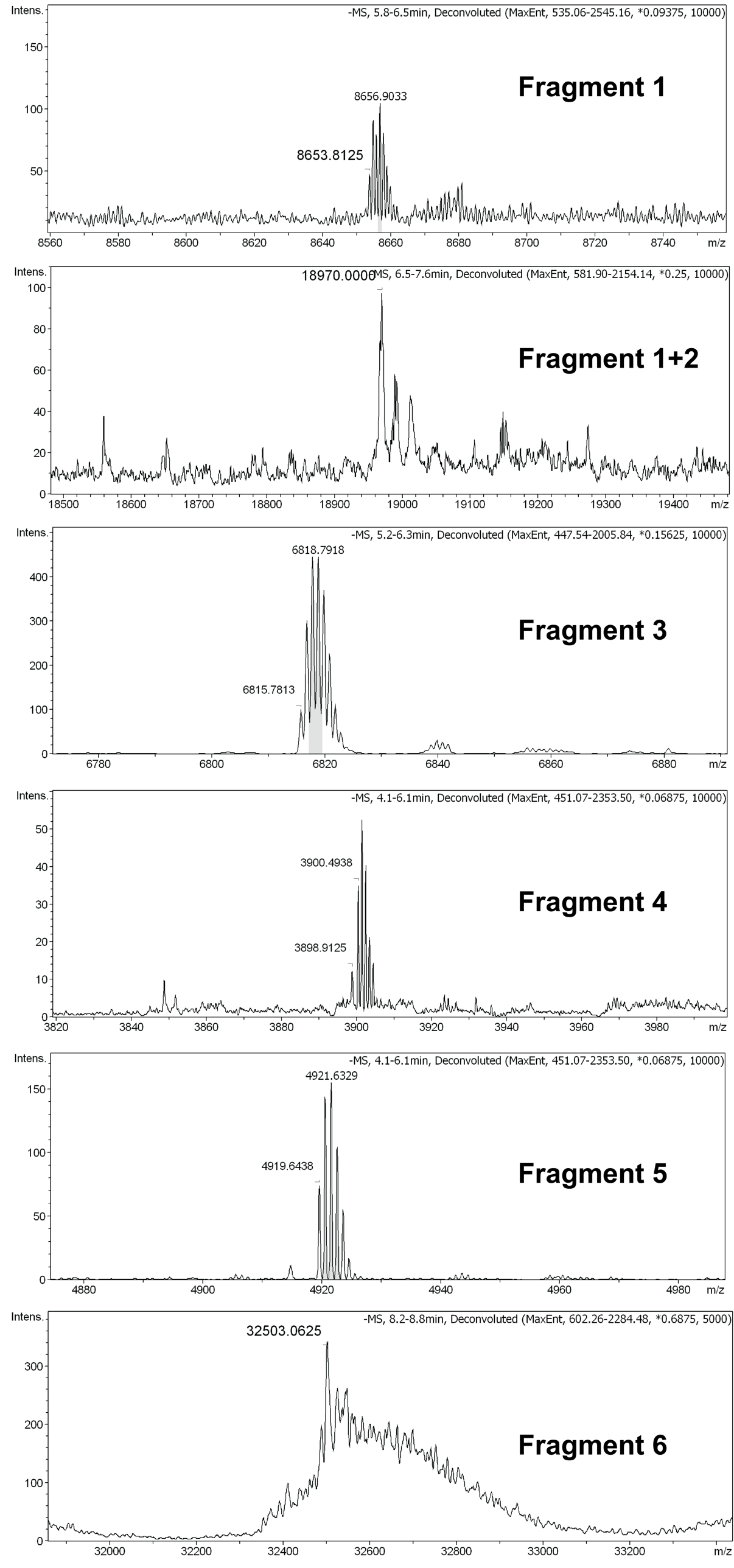
**

**Figure S12**. High-resolution LC-MS analysis of MazF cleavage products from *M215-025* mRNA. Deconvoluted mass spectra of individual RNA fragments generated by *E. coli* MazF endonuclease digestion, with observed masses and fragment assignments.

**

**

**Figure S13**. Alternative MazF cleavage products from *M215-native* RNA. Deconvoluted LC–MS spectra of noncanonical fragments S1–S3 generated by MazF cleavage at 5′-AAC-3′ motifs.

# **References**

1. Abe, N., Imaeda, A., Inagaki, M., Li, Z., Kawaguchi, D., Onda, K., Nakashima, Y., Uchida, S., Hashiya, F., Kimura, Y. *et al.* (2022) Complete Chemical Synthesis of Minimal Messenger RNA by Efficient Chemical Capping Reaction. *ACS Chemical Biology*, **17**, 1308-1314.

2. Ototake, M., Inagaki, M., Kimura, S., Onda, K., Tada, M., Kawaguchi, D., Murase, H., Fukuchi, K., Gao, Y., Kokubo, K. *et al.* (2024) Development of hydrophobic tag purifying monophosphorylated RNA for chemical synthesis of capped mRNA and enzymatic synthesis of circular mRNA. *Nucleic Acids Research*, **52**, 12141-12157.
